# Reading wild minds: A computational assay of Theory of Mind sophistication across seven primate species

**DOI:** 10.1371/journal.pcbi.1005833

**Published:** 2017-11-07

**Authors:** Marie Devaine, Aurore San-Galli, Cinzia Trapanese, Giulia Bardino, Christelle Hano, Michel Saint Jalme, Sebastien Bouret, Shelly Masi, Jean Daunizeau

**Affiliations:** 1 Université Pierre et Marie Curie, Paris, France; 2 Institut du Cerveau et de la Moelle épinière, Paris, France; 3 INSERM UMR S975, Paris, France; 4 Museum National d'Histoire Naturelle, UMR 7206, Paris, France; 5 Universita La Sapienza, Rome, Italy; 6 Ménagerie du Jardin des Plantes, Paris, France; Oxford University, UNITED KINGDOM

## Abstract

Theory of Mind (ToM), i.e. the ability to understand others' mental states, endows humans with highly adaptive social skills such as teaching or deceiving. Candidate evolutionary explanations have been proposed for the unique sophistication of human ToM among primates. For example, the *Machiavellian intelligence hypothesis* states that the increasing complexity of social networks may have induced a demand for sophisticated ToM. This type of scenario ignores neurocognitive constraints that may eventually be crucial limiting factors for ToM evolution. In contradistinction, the *cognitive scaffolding hypothesis* asserts that a species' opportunity to develop sophisticated ToM is mostly determined by its general cognitive capacity (on which ToM is scaffolded). However, the actual relationships between ToM sophistication and either brain volume (a proxy for general cognitive capacity) or social group size (a proxy for social network complexity) are unclear. Here, we let 39 individuals sampled from seven non-human primate species (lemurs, macaques, mangabeys, orangutans, gorillas and chimpanzees) engage in simple dyadic games against artificial ToM players (*via* a familiar human caregiver). Using computational analyses of primates' choice sequences, we found that the probability of exhibiting a ToM-compatible learning style is mainly driven by species' brain volume (rather than by social group size). Moreover, primates' social cognitive sophistication culminates in a precursor form of ToM, which still falls short of human fully-developed ToM abilities.

## Author summary

The contribution of Theory of Mind (ToM), i.e. the ability to understand others' mental states, to the cognitive toolkit of non-human animal species (including primates), is fiercely disputed. We contribute to this debate by (i) proposing a computational definition of ToM sophistication that is amenable to behavioural testing in non-human primates (which we had previously validated in humans), and (ii) performing a balanced comparison of seven primates species (from lemurs to monkeys to great apes). In turn, our study provides an unprecedented computational insight into the evolutionary roots of human social intelligence. In particular, we provide empirical evidence against the common-sense idea that sophisticated ToM evolved mostly as an "on-demand" response to social challenges posed by big herds. Rather, the evolution of sophisticated ToM seems to be mainly determined by neurobiological limiting factors such as the species' "cognitive reservoir". *En passant*, we identify an evolutionary gap between great apes and humans, in terms of the sophistication of their respective ToM skills.

## Introduction

How do you know what others think or feel? Theory of Mind (ToM), i.e. the ability to identify covert mental states from others’ overt behaviour, is a crucial component of human social intelligence. Although ToM endows humans with highly adaptive skills such as bonding, teaching or deceiving, its contribution to the cognitive toolkit of other animal species, including primates, is debated [1–3]. Thus, a few theories have been concurrently proposed as candidate explanations for why humans have evolved such unusually sophisticated ToM. For example, the "social brain hypothesis" posits that the complexity of primates' societies is the primary driver of primates' cognitive skills [4,5]. The existence of a statistical relationship across primate species between social group size (a proxy for social network complexity) and brain volume (a proxy for general cognitive capacity) is typically taken as evidence in support of this idea [6,7]. Critical here is the notion that the adaptive fitness of social cognitive skills may overcompensate the metabolic cost incurred by large brains [8,9] if the typical species' social organization is complex enough. Recent theoretical work demonstrated that such cost-benefit competition can explain the evolutionary dynamics of "Machiavellian intelligence" [10], i.e. a specific subset of cognitive skills geared towards achieving social success [11]. In short, sophisticated ToM would have evolved mostly as an "on-demand" response to social challenges posed by big herds. However, increases in brain volume may have arisen from other forms of selective pressure (e.g., unpredictable and dispersed food resources), eventually favouring non-social cognitive skills that endow primates with, e.g., innovative tool uses or foraging strategies [12–15]. In turn, the causal relationship may be reversed, i.e. larger brains may have eventually enabled species to build and maintain bigger social networks. Under this view, social intelligence is a byproduct of evolutionary pressure on brain volume, which has opened a window of opportunity for sophisticated ToM to emerge [16]. In other terms, the evolution of ToM would be mainly determined by neurobiological limiting factors such as the species' "cognitive reservoir" [17,18]. This idea is in line with developmental studies in humans that show that sophisticated ToM is, at least partially, "scaffolded" on domain-general cognitive improvement [19,20]. In what follows, we refer to this idea as the "scaffolding hypothesis" [4]. To date, discriminating between these evolutionary hypotheses has not been possible because it requires the difficult combination of (i) an operational definition of ToM sophistication that is amenable to behavioural testing in non-human primates, and (ii) a balanced comparison of ToM sophistication in primate species that differ in terms of sociobiological features such as group size and brain volume. These are the issues we address in this work, using combined experimental and computational means.

Most non-human primates typically engage in diverse and complex social interactions, exhibiting seemingly deceptive and manipulative behaviour [21,22]. Following early experimental investigations [23], positive evidence has supported the idea that chimpanzees—arguably the smartest non-human primate species and the phylogenetically closest to humans- understand what conspecifics know [24], want [25] or learn [26]. This line of investigation, however, has been challenged by negative results regarding, e.g., the ability to understand what others perceive [27–29] or to distinguish between one’s own belief and others’ [30–32]. In retrospect, positive evidence might simply have neglected simpler behaviorist explanations of animal policies in social contexts, such as flexible forms of stimulus-response associative learning [2]. Furthermore, notwithstanding a few recent studies on non-ape species—mostly about macaques or other old world monkeys—yielding similarly inconsistent results [33–36], no systematic comparative study of ToM across primate species has been conducted. This eventually raised profound methodological and theoretical concerns regarding theories of ToM's evolutionary foundations based on existing ethological studies [2,37–40].

Taking inspiration from recent advances in machine learning and cognitive psychology [41,42] we suggest an operational definition of ToM that departs from previous qualitative ToM investigations. We start with the premise that ToM solves a specific evolutionary challenge, namely: predicting others' overt behaviour from learned associations with social cues (including past behaviour). Critical here is the notion that primate species may differ with respect to their *learning styles*, whose sophistication may depend upon their innate cognitive structure [16]. Arguably, somewhere at the end of the spectrum lie human learning styles that derive from so-called metarepresentational ToM [43], whose sophistication increases with the depth of recursive beliefs (as in "I believe that you believe that I believe…"). These highly sophisticated forms of ToM possess adaptive value in the context of strategic social interactions, in which individuals can learn about each other [44–46]. Nevertheless, learning in such contexts can take less sophisticated forms, ranging from simple heuristics, to trial-and-error learning, to cognitive precursors of ToM that simply care about others' overt reaction to one's own actions [47]. Critically, mathematical modelling can be used to turn a given learning style into a learning rule (i.e. the precise way in which agents adapt to the history of past actions and feedbacks), whose cognitive sophistication is formally defined in terms of the computational complexity of information processing [48]. In appropriate experimental contexts (e.g., dyadic games), this endows learning styles with a specific behavioural signature that can be disclosed from quantitative analyses of trial-by-trial choice sequences. In turn, the cognitive sophistication of learning styles can be inferred from observed overt behaviour, and eventually compared across species. We have previously validated this computational approach by showing that when engaging in mentalizing, human adults’ learning styles are specifically captured by second-order recursive belief updating schemes [49]. We now extend this approach to a comparison of non-human primate species, and ask which of the above hypotheses is the most likely explanation for the evolution of social intelligence.

**Fig 1:**
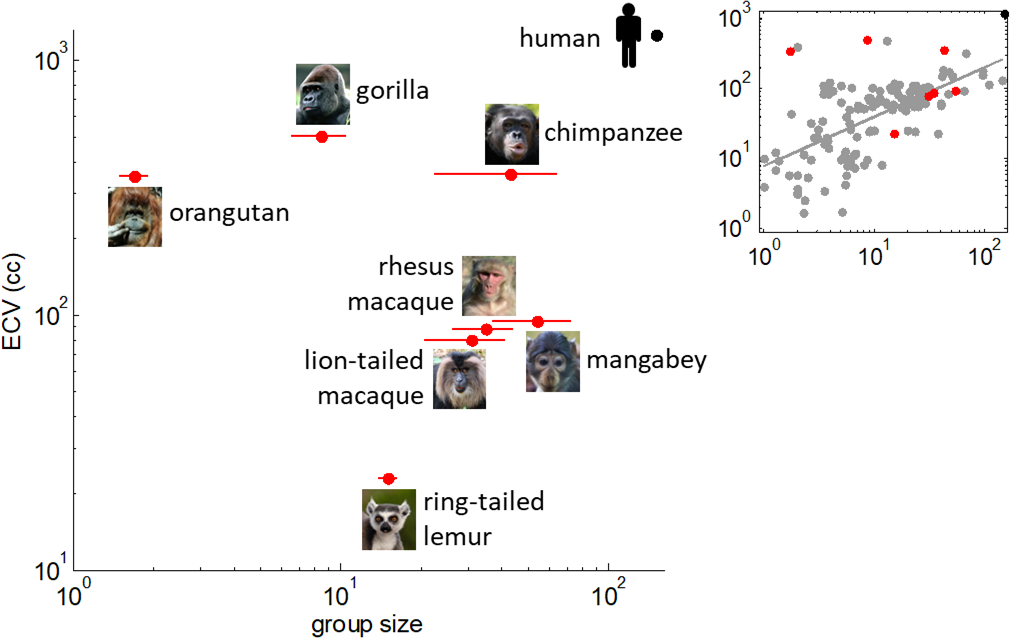
Sociobiological features of tested non-human primates species. On this graph, the social group size (x-axis) and ECV (y-axis) are shown for each species on a log-log scale. Note that reported species' group sizes exhibit substantial variability across ethological field studies. In this work, we have chosen to rely on the average group size from a series of more than a hundred published studies (ensuing standard deviations are depicted as horizontal bars on the graph). We refer the interested reader to S2 Text for more details. Overall, there is no significant statistical correlation between group size and ECV (r = -0.37, p = 0.41) across these species. The position of the human species is shown for comparison purposes. The graphical inset also shows the relationship between group size and ECV, this time across the 130 primate species reported in [91]. Species investigated in this work are depicted in red.

We let 39 individuals from seven non-human primate species with different phylogenetic distances from humans (including lemurs, macaques, mangabeys, orangutans, gorillas and chimpanzees) play simple repeated games with familiar zookeepers who followed the instructions of (on-line) learning algorithms endowed with calibrated ToM sophistication. Fig 1 below depicts the statistical relationship between endocranial volume (ECV) and social group size (in the wild) across primate species. Critically, although ECV and social group size are correlated across the full range of primate species (r = 0.62, p<10^−4^; see graphical inset in Fig 1), the sample correlation is very weak across the seven tested species (r = -0.37, p = 0.41; see Fig 1). This enables us to evaluate the evidence for candidate evolutionary scenarios by identifying the ensuing statistical relationships existing between social group size, brain volume and ToM sophistication, across tested species. Note that there is an ongoing debate regarding which sociobiological feature of primate species is appropriate for such type of analysis (see first section of S1 Text). We will comment on this and related issues in the Discussion section.

## Results

**Fig 2:**
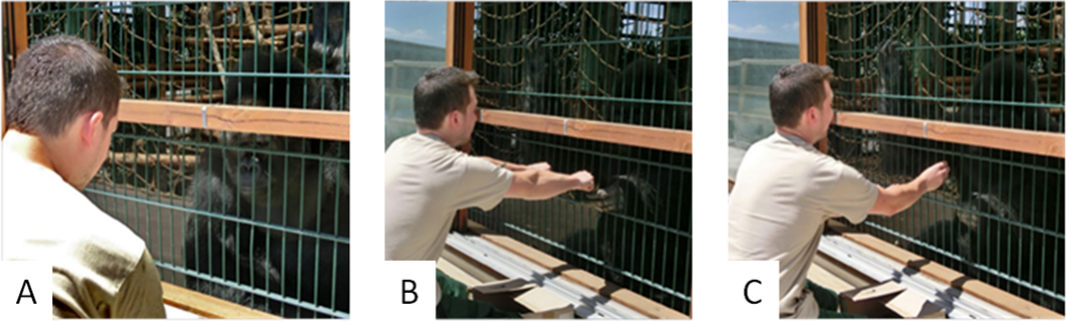
Experimental protocol showing the three basic phases of the game. **A**: the experimenter hiding the food in one hand out of individual’s view (inside the brown paper box visible in two following pictures), **B**: the individual choosing one hand by pointing or touching it, **C**: the individual getting the food reward if choosing the correct hand. Photo credits C. Trapanese.

Our main task consisted of multiple sessions of a so-called "hide and seek" game (60 trials each) against three distinct opponents (below). To succeed, primates had to anticipate and predict the behaviour of their opponent, who hid a fruit in one out of two possible locations (left/right hand) at each trial (see Fig 2 below). Opponents either followed a predetermined pseudo-random sequence with a 65% bias for one hand (condition *RB*), or attempted to deceive the primates from learned anticipations of their behaviour (conditions *0-ToM* and *1-ToM*). The difference between *0-ToM* and *1-ToM* lies in how they learn from the past history of primates’ actions. In brief, *0-ToM* does not try to interpret the primates' action sequence in terms of a strategic attempt to win. Rather, it simply assumes that abrupt changes in the primates' behaviour are a priori unlikely. It thus tracks the evolving frequency of primates’ actions, and chooses to hide the reward where it predicts the primate will not seek. It is an extension of “fictitious play” learning [50], which can exploit primates' tendency to repeat their recent actions. In contrast, *1-ToM* is equipped with (limited) artificial mentalizing, i.e. it attributes simple beliefs and desires to primates. More precisely, it assumes that primates’ actions originate from the strategic response of a *0-ToM* agent that attempts to predict his own actions. Note that the computational sophistication of artificial mentalizing is not trivial, since *1-ToM* has to explicitly represent and update its (recursive) belief about its opponents' beliefs. In turn, *1-ToM* learning essentially consists in an on-line estimation of *0-ToM*’s parameters (i.e.: learning rate and behavioural temperature; see Methods) given the past history of both players’ actions. This makes *1-ToM* a so-called “meta-Bayesian” agent [49,51] that can outwit strategic opponents that do not mentalize when competing in the game (such as *0-ToM*). Critically, primates were not cued about opponent conditions. This implies that they had to adapt their behaviour according to their understanding of the history of past actions and outcomes. In addition, except in the control (*RB*) condition, there is no possibility to learn the correct answer from simple reinforcement. This is because *0-ToM* and *1-ToM* artificial learners exhibit no systematic bias in their response. Further details regarding the experimental protocol (including animal training) as well as *k-ToM* artificial agents can be found in the methods section below.

**Fig 3:**
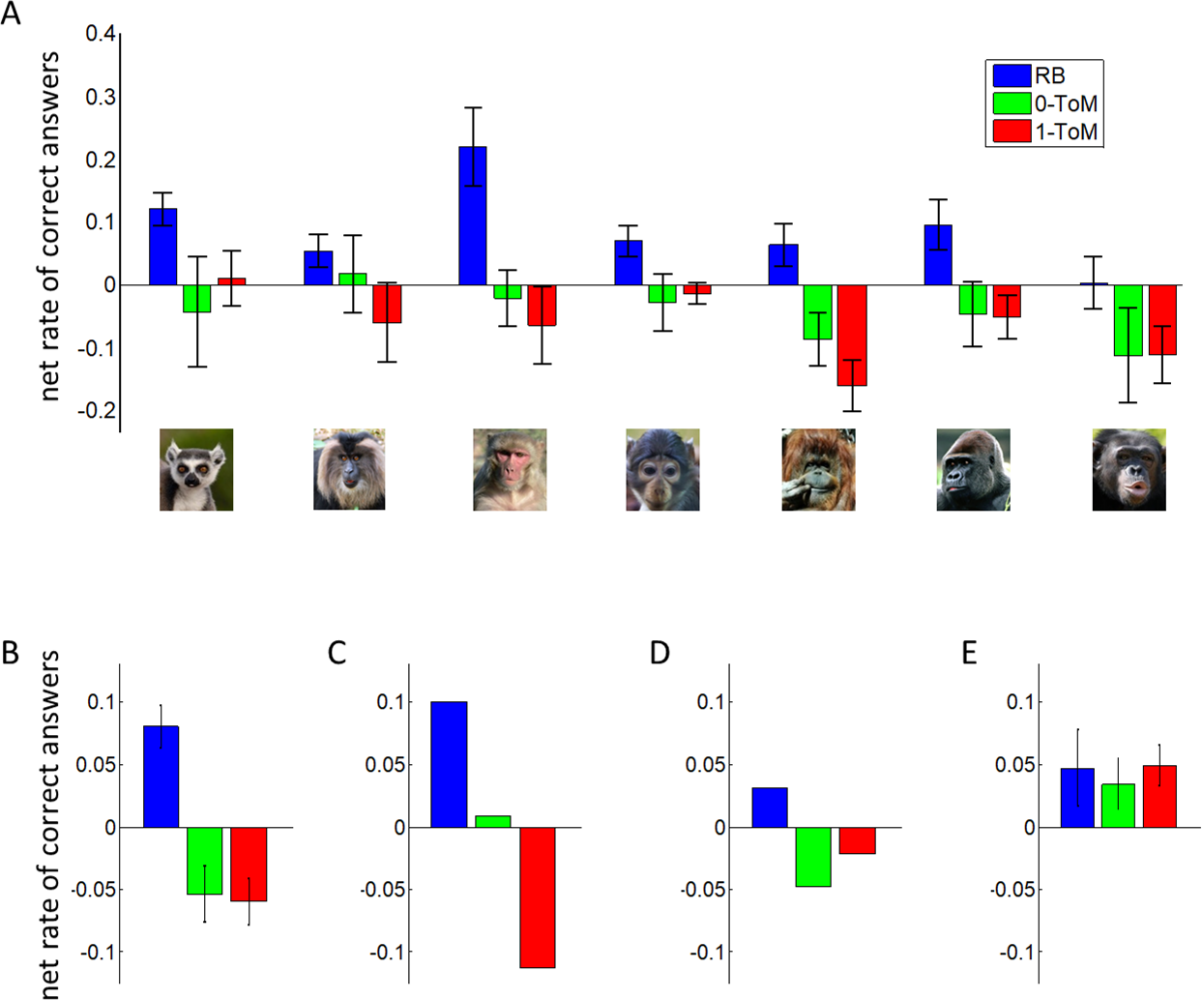
Behavioural performance results. **A:** net rate of correct answer (y-axis) is shown as a function of species (x-axis) and opponent condition (*RB*: blue, *0-ToM*: green and *1-ToM*: red). Errorbars depict standard error of the mean. **B:** condition-specific performance pattern averaged across species. **C:** simulated performance pattern of 0-ToM. **D:** simulated performance pattern of cooperative 1-ToM. **E:** performance pattern of human adults [49]. Graphs B to E use the same colour coding as in A.

As we will see below, one cannot unambiguously recognize primates' ToM sophistication from their pattern of performance across task conditions. Rather, one has to decompose action sequences and identify learning styles. Nevertheless, let us start with a simple summary of performance results. Fig 3A below shows the net rate of correct answers (averaged across individuals within species), after adjustment for non-specific session effects (see Methods section).

One can see that, on average, primates seem to perform reasonably well in the control condition (*RB*), which means that they have understood the basic tasks' rules. We performed a random-effect analysis to test for the effects of opponent' sophistication and species onto performance (see Methods). At the group-level, we found a significant main effect of opponent (F[2,58] = 14.0, R^2^ = 32.6%, p<10^−4^) and a trend for a main effect of species (F[6,58] = 2.17, R^2^ = 18.3%, p = 0.06). No interaction between species and opponent was found (F[12,58] = 1.0, R^2^ = 17.1%, p = 0.46). Moreover, when further testing inter-species differences, we found that the ECV predicted overall performance (F[1,58] = 5.2, R^2^ = 32.6%, p = 0.026) whereas group size did not (F[1,58] = 0.3, R^2^ = 0.5%, p = 0.14). Intriguingly, the effect of ECV went in the opposite direction of what could be intuitively expected, in that having a larger brain actually yields worse performance on average. As will be clearer below, this is due to the non-trivial effect of ToM sophistication on performance in this task. This issue will be addressed later, using model-based analyses of action sequences. Now eyeballing the opponent's effect on performance (see Fig 3B) reveals the following pattern: overall, primates win in the control (*RB*) condition, whereas they tend to lose similarly against *0-ToM* and *1-ToM*. This strongly contrasts with the results of our previous experiment on healthy human participants [49], who win against *0-ToM* and *1-ToM*, most likely by relying on sophisticated mentalizing akin to competitive *2-ToM* learning (see Fig 3E). In fact, two classes of learning styles would be qualitatively compatible with the pattern of primates’ performances across conditions. On the one hand, numerical simulations show that simple non-mentalizing learning schemes such as *0-ToM* show a gradual performance decrement with opponent's sophistication (see Fig 3C). On the other hand, cooperative strategies based upon sophisticated mentalizing (e.g., *1-ToM* or *2-ToM*) eventually win against *RB* and lose against *0-ToM* and *1-ToM* (see Fig 3D). Thus, discriminative evidence for or against mentalizing can only be derived from quantitative analyses of trial-by-trial choice sequences. As we will see, these are in fact much more sensitive and informative than model-free performance analyses.

**Fig 4:**
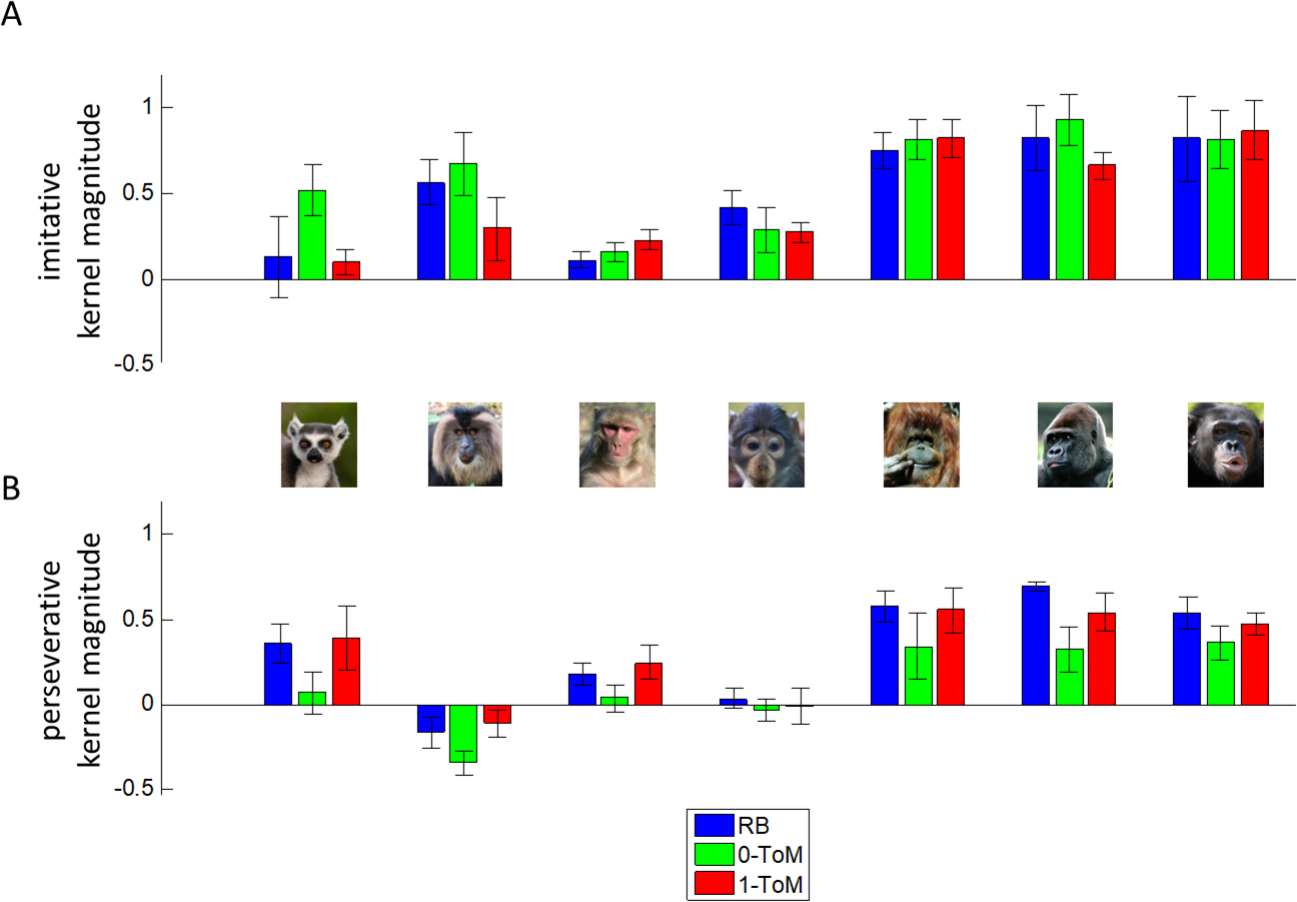
Volterra decomposition of primates’ trial-by-trial choices sequences. The magnitude of Volterra kernels (y-axis) is plotted as a function of species (x-axis) and opponent condition (same colour coding as in Fig 3). **A:** weight of the opponent’s actions (imitative tendency). **B:** weight of one’s own actions (perseverative tendency).

Our second step of analysis thus consisted of Volterra decompositions [52] of primates' choice sequences, i.e. we looked at how much trial-by-trial variance in choice sequences can be concurrently explained by the past history of both players actions (see Methods). This decomposition enables us to capture learning styles in terms of model-free mixtures of imitative and perseverative tendencies [49]. Fig 4 below summarizes the mean magnitudes of each species' Volterra kernels, for all conditions.

One can see that, on average, primates tend to imitate their opponents' choices (positive impact of past opponent's choice, *A*^*op*^), which is a good strategy when playing against *RB* because this, on average, yields reward more often than chance. Although this is reminiscent of a "win-stay/lose-switch" heuristic strategy, we will see below that other learning styles may eventually exhibit this tendency. In addition, they also seem to perseverate, i.e. they tend to repeat their own past choices (positive *A*^*self*^ on average). However, the relative magnitudes of imitative and perseverative tendencies seem to differ across species and conditions. Thus, we performed a random-effect analysis to test for the effects of opponent' sophistication and species onto perseverative (*A*^*self*^) and imitative (*A*^*op*^) tendencies. We found a main effect of opponent for *A*^*self*^ (F[2,58] = 9.8, R^2^ = 25.3%, p = 2×10^−4^) but not for *A*^*op*^ (F[2,58] = 2.3, R^2^ = 7.3%, p = 0.1). This is important, since this is a sign of a (moderate) strategic adaptation to opponents, such that primates persevere less against *0-ToM* than in the other conditions. In addition, we found a strong effect of species on both *A*^*self*^ (F[6,58] = 22.0, R^2^ = 69.5%, p<10^−4^) and *A*^*op*^ (F[6,58] = 19.0, R^2^ = 66.3%, p<10^−4^), and no interaction (p = 0.7 for *A*^*self*^ and p = 0.5 for *A*^*op*^). Note that, when further investigating inter-species differences, we found that both imitative and perseverative tendencies increased with EVC and network size (all p<10^−4^). At this point, we asked whether the effect of species and opponent onto performance were mediated by changes in learning styles. We thus computed the correlation between the estimated Volterra kernel of each individual's choice sequence in each condition and that of the corresponding optimal learning style (namely: 0-ToM against *RB*, 1-ToM against *0-ToM* and 2-ToM against *1-ToM*). Classical Sobel mediation tests [53] then confirmed that primates' similarity to optimal learning styles mediated the effect of opponent (p = 0.025), ECV (p = 4×10^−4^) and group size (p = 5×10^−4^) onto performance. We refer the interested reader to the Methods section for methodological details regarding Volterra analyses.

**Fig 5:**
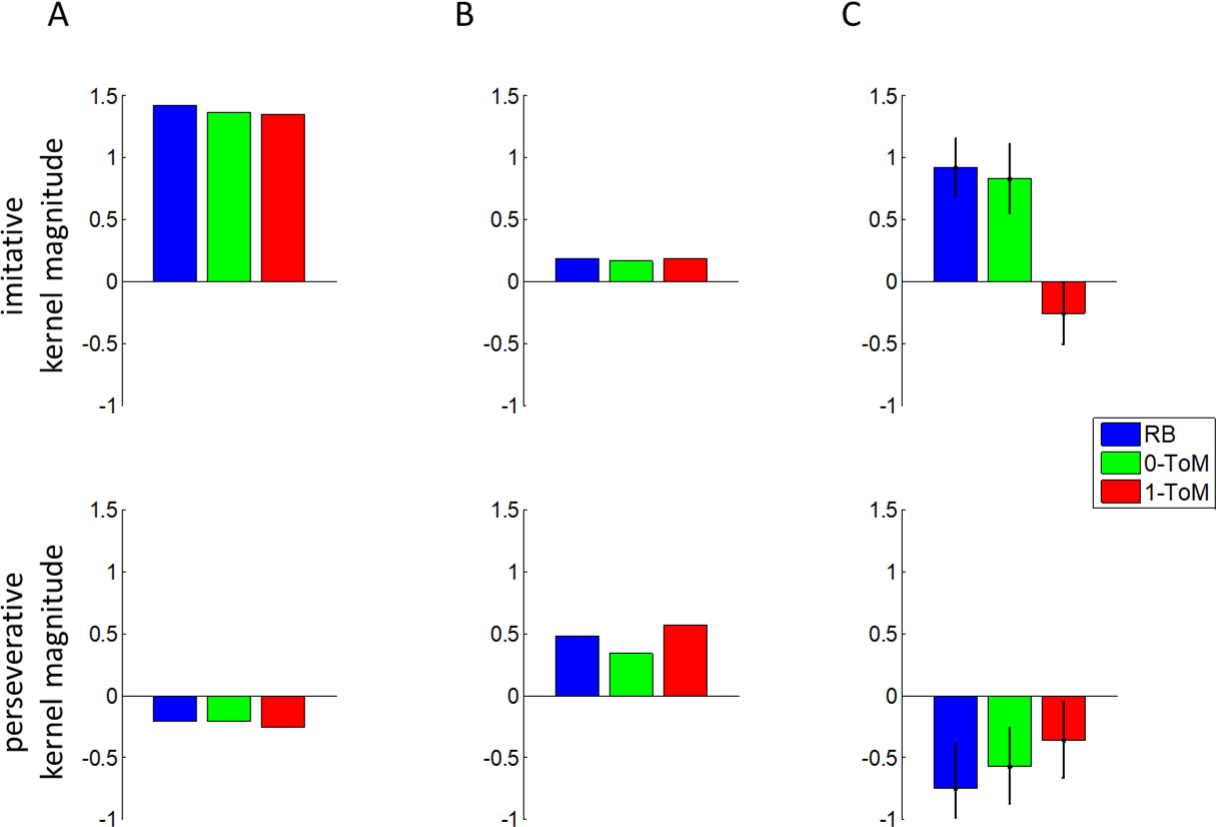
Volterra decompositions of non-mentalizing and cooperative mentalizing artificial agents, as well as human adults [49] performing the same task. **A:** competitive 0-ToM, **B:** cooperative 1-ToM, **C:** human adults. Same format as Fig 4.

These results are important, because they indicate that performance variations are likely to be driven by differences in species-specific learning styles. For example, a tendency to perseverate may signal a strategic behavioural response relying on sophisticated ToM inference, based on a cooperative interpretation of the game. Intuitively, if primates believe that the goal of the zoo keeper (the opponent) is aligned with their own (e.g., that he wants to feed them), then repeating their own choices is instrumental (it serves the purpose of achieving coordination). Fig 5 below illustrates how different the Volterra kernels of cooperative learning styles and non-mentalizing learning styles can be. We also included a summary of the Volterra results from our previous experiment in humans, which will serve as a reference point.

To begin with, note how Volterra kernels of human subjects differ from those of non-human primate species. Critical here is the fact that they adapt their imitative and perseverative tendencies in a quasi-optimal manner. In particular, humans correctly repress their imitative tendency when playing (unknowingly) against 1-ToM (as competitive 2-ToM learners do). No non-human primate species exhibits such adaptive flexibility. As one can see on Fig 5, primates' Volterra kernels are in fact more compatible with either non-mentalizing agents (0-ToM) or cooperative agents with mild sophistication (1-ToM). More precisely, the strong and rigid imitative tendency of most primate species is similar to 0-ToM’s, while the moderate flexibility of their perseverative tendencies is rather reminiscent of cooperative 1-ToM learning (cf. U-shaped perseverative kernels across opponent conditions).

Taken together, we have found strong inter-species differences in Volterra kernels, and some of these variations may be compatible with mentalizing learning styles. One cannot, however, directly interpret quantitative changes in Volterra kernels across species in terms of differences in, e.g., cooperativeness or learning style. Evidence for the latter can only be derived from direct quantitative comparisons of primates’ trial-by-trial choices sequences and predictions derived from learning models. In what follows, we report the results of a statistical (Bayesian) model comparison that quantifies, for each species, the evidence in favour or against ToM-compatible learning styles, given primates' trial-by-trial choice sequences.

**Fig 6:**
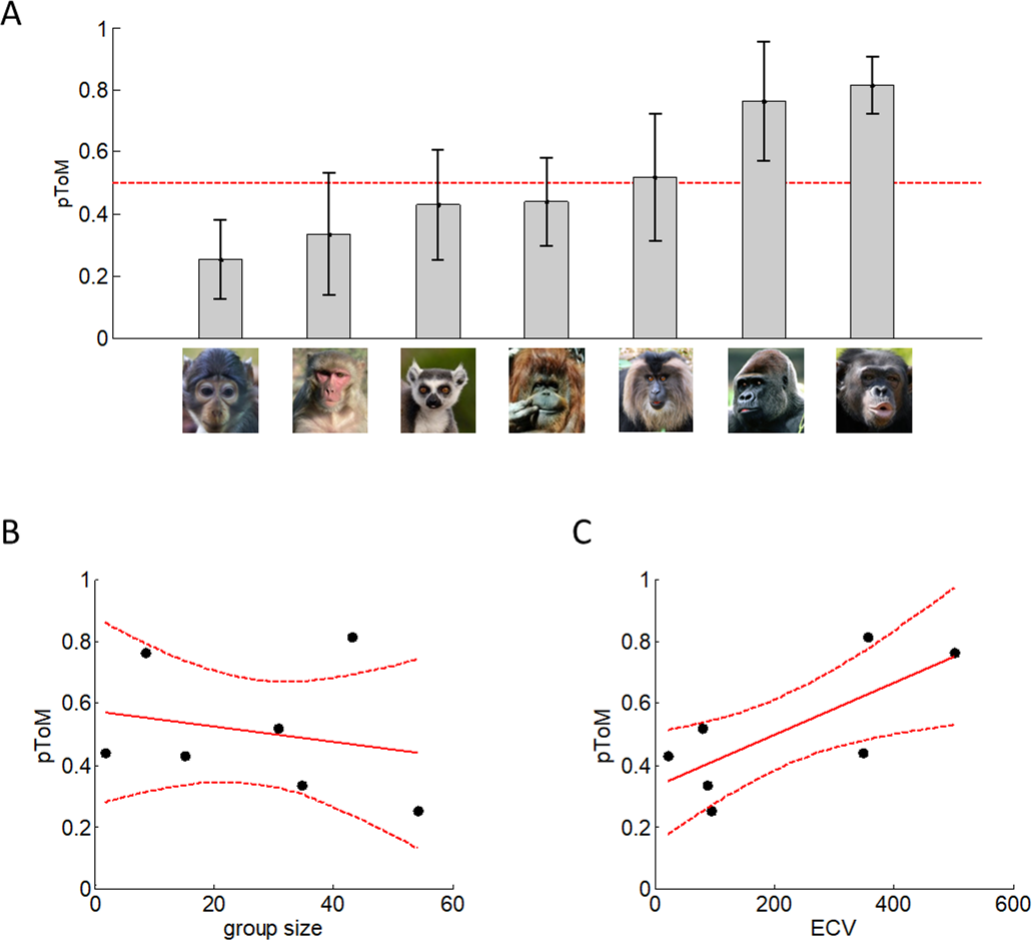
Bayesian model comparison results. **A**: the average probability *pToM* of exhibiting a ToM-compatible learning style (±standard error) is plotted for each species, in ascending order. The red dotted line corresponds to chance discrimination (*pToM* = 0.5). Note that orangutans' ToM sophistication reaches *pToM* = 0.51±0.14 if we exclude one individual that shows characteristic signs of Down syndrome (see Discussion section). **B:** the probability of exhibiting a ToM-compatible learning style (*pToM*, y-axis) is plotted as a function of group size (x-axis). The red plain line indicates the best-fitting linear regression, and the red dotted lines depict the corresponding 95% confidence interval. **C:** the probability of exhibiting a ToM-compatible learning style (*pToM*, y-axis) is plotted as a function of ECV (x-axis).

We considered a set of candidate learning models that differ in terms of their sophistication, ranging from simple behavioural heuristics, to mildly sophisticated learning schemes, to ToM-based (meta-Bayesian) recursive belief update schemes. This model set first consists of a family of four different non-ToM models, namely: *BN* (biased Nash), *WS* ("win-stay/lose-switch" heuristic), *RL* (reinforcement learning) and *0-ToM*. In addition, we included a family of six ToM models, namely: *Inf* (cooperative and competitive "influence learning"), *1-ToM* (cooperative and competitive) and *2-ToM* (cooperative and competitive). Each of these computational models provides a probabilistic prediction of observed primates' trial-by-trial choice sequences, given the past history of players' actions and specific unknown parameters controlling e.g., biases and learning rates [49]. Note that the essential difference between ToM and non-ToM models is that only the former assume that observed responses are intentional actions. We fitted these models on primates’ trial-by-trial choice sequences and evaluated their marginal likelihood. We then derived a species-specific estimate of the probability *pToM* of exhibiting a ToM-compatible learning style. We refer the interested reader to the Methods section for details regarding computational models and the ensuing statistical model comparison procedure, the result of which is summarized on Fig 6 below.

We are now in a position to directly compare our two main hypotheses. Recall that under the *Machiavelian intelligence hypothesis*, ToM sophistication should mostly align with social group size, whereas, under the *cognitive scaffolding hypothesis*, it should rather align with brain volume (ECV). We can directly test these predictions by asking whether inter-species differences in *pToM* are best predicted by either group size or brain volume. The result of this procedure is summarized on Fig 6 below.

Fig 6A reports the estimated probability of exhibiting a ToM-compatible learning style (pToM). One can see that this probability varies greatly across species, ranging from pToM = 0.25 ± 0.12 (mangabeys) to pToM = 0.81 ± 0.09 (chimpanzees). Fig 6B summarizes the statistical relationship between group size and pToM (across species). One can see that the pairwise correlation between the two variables is very weak and does not reach statistical significance (r = -0.22, p = 0.69). Now Fig 6C summarizes the statistical relationship between ECV and pToM. Here, there is a strong and significant pairwise correlation between the two variables (r = 0.75, p = 0.03). Note that this result remains statistically significant when accounting for the structured phylogenic relationships between these species (p = 0.04 for a one-sided test on the correlation; cf. S1 Text). In addition, ECV is marginally better than group size at predicting inter-species variability in pToM (p = 0.07). These qualitative results are left unchanged if one assumes that inter-species variability in pToM results from a linear mixture of inter-species variability in group size and ECV. Indeed, when regressing pToM concurrently against both ECV and group size, we find that the effect of ECV is significant (t[4] = 2.18, adjusted R^2^ = 54.3%, p = 0.047) whereas group size is not (t[4] = 0.28, adjusted R^2^ = 2.0%, p = 0.39). This holds true even if we account for the interaction between ECV and group size (ECV: p = 0.02, group size: p = 0.45, [ECV x group size]: p = 0.13), or if we include the human species in the analysis (ECV: p = 0.02, group size: p = 0.95; assuming pToM[humans] = 1).

**Fig 7:**
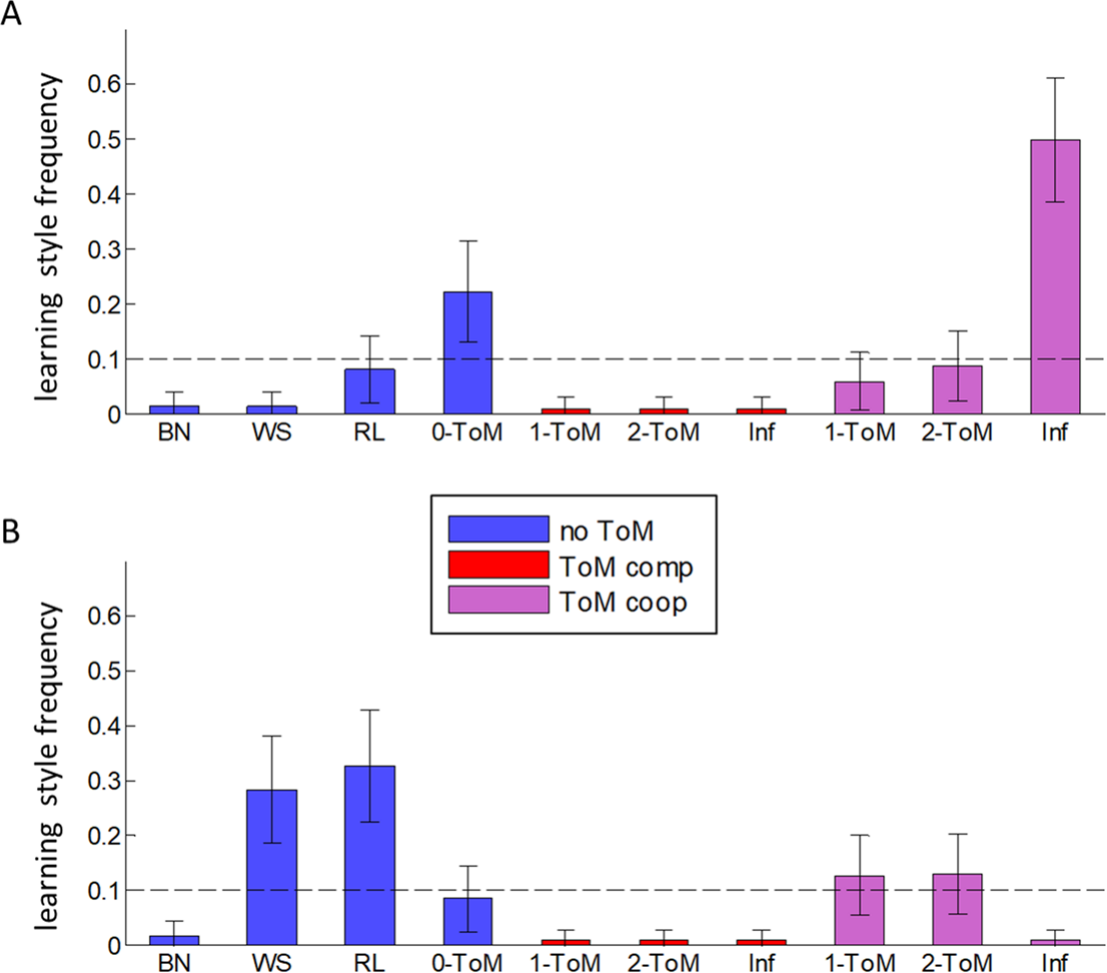
Estimated frequencies of learning styles. The posterior mean of model frequency (y-axis) is shown for each learning style (x-axis), among species with large brains (**A**) and small brains (**B**). Note that the median-split on ECV actually separates apes from prosimians and monkeys, which is consistent with primates’ phylogenic relationships (see S1 Text). The colour code indicates the type of learning style (blue: no-ToM, red: competitive ToM, violet: cooperative ToM). Errorbars indicate posterior standard deviations. For comparison purposes, chance level (10%) is indicated using black dotted lines.

Let us now ask which learning style (among the ten candidate models considered here) best captures choice sequences within species with either small or large brains (according to a median-split on ECV). Note that, using a between-groups Bayesian model comparison [54], we find that the posterior probability that species with large brains have evolved a more ToM-sophisticated learning style than species with small brains is P = 0.99. Additional details regarding this procedure can be found in S1 Text. Fig 7 below shows the estimated frequency of all learning models for each subgroup of species.

One can see that the two subgroups of species strongly differ in terms of learning styles prevalence. More precisely, the learning style that best captures choice sequences of primate species with large brains is the cooperative "influence learning" model (estimated frequency = 50%), whereas species with small brains seem to mostly rely on either reinforcement learning (estimated frequency = 33%) or "win-stay/lose-switch" strategies (estimated frequency = 28%). These results are qualitatively consistent with the previous model-free analyses, essentially because "influence learning" exhibit performance and Volterra patterns that are similar to those of *1-ToM*. Importantly, none of these subsets of species matches our previous estimate of human ToM sophistication, which was dominated by *2-ToM* learning styles [49]. This signals an evolutionary gap between apes and humans, given that "influence learning" is much less sophisticated than *2-ToM* learning. We will comment on the computational distinction between "influence learning" and *k-ToM* learning in the discussion section below.

## Discussion

In this work, we have performed a comparison of non-human primate species playing simple competitive games against human opponents. Using computational analyses of primates' choices sequences, we found that inter-species differences in ToM sophistication are predicted by differences in brain volume but not by differences in social group size. Moreover, we identified an evolutionary gap between great apes and humans, in terms of the sophistication of their respective ToM skills.

Our results provide evidence against the common-sense notion that selective pressure favoured sophisticated ToM in species that lived in bigger herds. They are in line with studies showing that, e.g., the prevalence of social learning (e.g., imitative behaviours) is correlated with neocortex ratio but not with social group size [14]. This immediately raises the following question: given the biological cost of brain tissue, what then endows social intelligence and, in particular, ToM sophistication, with adaptive fitness? One possibility is that, when it comes to comparing social cognitive skills, social group size is a poorly reliable proxy for the complexity of primates' societies. This has led some authors to rather focus on field reports of, e.g., "animal culture", which would be operationally defined as the within-species heterogeneity of socially-acquired behaviour [55]. Alternatively, the adaptive fitness of ToM sophistication may depend in a non-trivial manner on the nature of within-species interactions. For example, it has been shown, using evolutionary game theory, that cooperative interactions promote ToM sophistication to a much lesser extent than competitive interactions, essentially because less sophisticated phenotypes can benefit from the sophistication of cooperative partners [44]. Yet another perspective is that complex primate societies may endow ToM with adaptive fitness only when in conjunction with other socially-relevant skills such as, e.g., intentional communication [56,57], empathy [58] or reputation management [59]. None of the above suggestions actually challenge the idea that ToM has been selected because it addressed some of the specific challenges posed by complex (primate) societies. But this neglects the fact that, in most primate species, combinations of rigid social norms and/or hierarchies with mundane though expedient heuristics have proven sufficient to solve most social challenges [60–63]. An intriguing alternative however, is that sophisticated ToM derived its adaptive fitness from its contribution to solving *non-social* challenges. For example, social skills such as ToM may enable group members to "distribute their cognition by storing information into other minds" [4,64]. Humans, in particular, have reached an unprecedented level of "distributed cognition", anecdotally culminating in unique forms of collective memory [65]. Under this view, if equipped with the "cognitive reservoir" necessary to scaffold sophisticated ToM, a species can bypass the cognitive limitations of its constituent individuals. Although highly speculative, this perspective is interesting because it explains how a moderate though critical ToM gap between apes and humans can eventually trigger the remarkable evolutionary success of the human species [39]. We will further discuss this notion on computational grounds below.

Let us now discuss a few striking aspects of our inter-species comparison. This work corroborates the existing body of studies that provide evidence for a rudimentary form of ToM in apes, as opposed to prosimians and monkeys [16,35,66]. This is perhaps best exemplified on Fig 7, which shows the relative frequencies of learning styles for apes and monkeys, respectively: the former learn the influence they may have on others, whereas the latter engage in some form of trial-and-error learning heuristics. Our results are in line with field studies reporting that, e.g., monkeys show some evidence of imitative behaviours, but to a much lesser extent than apes [67,68]. This resonates with the quote that "apes are good psychologists—in that they are good at reading minds—whereas monkeys are good ethologists—in that they are good at reading behaviour—" [69]. Note that one may be surprised by the relatively disappointing results of orangutans, whose estimated ToM sophistication does not quite live up to one's expectations. This deserves a few clarifying comments. First, our estimate of orangutans' ToM sophistication (worse than other apes but better than most monkey and prosimian species) may in fact be deemed quite consistent with what would be expected from their position in the primates' phylogenic tree (see S1 Text). Second, there is in fact very few published studies on orangutans' ToM, and these yield quite inconsistent results [30,70,71]. Third, we tested orangutans in different zoos. This implies that tested individuals are not coming from a single population, which increases the chance that our results are generalizable. Finally, one of the orangutans is somehow special in that she is showing characteristic signs of primates’ Down syndrome [72]. Interestingly, her *pToM* score is zero, which may have decreased our empirical estimate of orangutans ToM sophistication (*pToM* = 0.51±0.14 if this individual is excluded). Note that the results of our analyses are left qualitatively unchanged if we exclude this individual from the data sample.

Recall that our experiment aimed at revealing the sophistication of learning styles by observing the patterns of primates' response to the history of choices from artificial agents endowed with calibrated ToM sophistication. We had originally designed the experiment using competitive agents mostly because it yielded the best expected discriminability between learning styles [44,49]. However, despite careful training sessions (see methods), primates seem to have partly misinterpreted the human opponent's intentions. In particular, those primate species that display a ToM-compatible learning style behave as if they were engaging in a cooperative game. This may be seen as an unavoidable consequence of the fact that primates were playing with their usual (human) caregivers, who are feeding them on a daily basis. One may thus wonder whether this non-ecological aspect of our experimental paradigm may have influenced our analyses. For example, one may think that this may have somehow impeded on their pragmatic understanding of the task. However, primates perform well above chance level against *RB*, which indicates that they have at least understood the game's contingency between their choice and the reward they get. In fact, primates also perform *below* chance level against *0-ToM* and *1-ToM*, which should count as evidence that their learning style was consistent enough to be exploited by artificial competitive agents.

On a similar line, one could argue that observed inter-species differences may be confounded by variations in domain-general cognitive competence, which would eventually determine learning efficiency. The intuition here is that, with sufficient training, animals could eventually learn the best response to their opponent, without having to mentalize. We agree that this is in principle possible, since *k-ToM* artificial agents are reducible (up to about 80% accuracy) to a linear convolution of past competing players' actions [49]. Thus, known specificities of species cognitive skills (such as, e.g., working memory or attention) could in principle make a difference. To begin with, note that our stopping criterion for the training/habituation phase was based upon performance, i.e. all species engaged the main protocol with an identical understanding of the task (see methods section below). Now, irrespective of any potential performance improvement across session repetitions, the evidence in favour of ToM-compatible learning styles correlates *negatively* with performance (cf. main effect of ECV). Finally, in contrast to Volterra kernel magnitudes, we found no difference in Volterra decay rates across species. This means that the effective number of past trials that was impacting on subjects' behavioural responses was the same for all species. In other terms, all species learned from the same amount of past remembered/attended actions and outcomes, but they differed in *how* they learned. Taken together, this makes domain-general cognitive competence an unlikely confounding factor for our computational results.

One may also question the robustness and/or efficiency of our computational approach. First, recall that Bayesian inference is immune to the statistical criticisms that have been raised against the use of p-values in classical inference [73–75]. Nevertheless, one may wonder whether our model-based Bayesian data analysis may not be somehow biased towards ToM-compatible models, eventually yielding artefactual results. This is highly unlikely however, given the differences in model comparison results for species with small and large brains (cf. S1 Text). In brief, it is difficult to think of a statistical bias (favouring either more or less sophisticated models) that would be inconsistently expressed in two different groups of subjects. Second, one may ask how reliable our model-based results are, given the apparent complexity of the Bayesian statistical procedure. Beyond authoritative arguments, we are committed to provide pragmatic demonstrations of our methodological rigor. First, we performed a statistical confusion analysis, which confirmed that candidate models were well identifiable under our experimental design (see S1 Text for details). This means that the potential algorithmic imperfections of our statistical procedure do not compromise the interpretation of our results. Second, although less sensitive, the results of performance and Volterra analyses are consistent with our model-based conclusions (cf. Figs 4 and 5). This provides construct validity to our computational approach. Finally, one may argue that our sample of selected species is too small for drawing any definitive conclusion. We acknowledge that, in statistical terms, our sample size is arguably limited (n = 7 primate species and about 5 individuals per species). However, it is largely exceeding the standards in the field, in which data availability is a known issue [76,77]. Besides, it is in fact remarkable that we detect our effect of interest in the context of such small-powered study.

Equipped with computational means for discriminating learning styles, we have separated learning styles that do rely on mentalizing from learning styles that do not. This effectively induced some sophistication cut-off between those behavioural patterns that are likely to be based upon ToM and those that are not. We used this to assess the evidence in favour of a statistical relationship between ToM sophistication and either brain volume or group size. This raises a number of related comments.

First, one may ask how robust to changes in species' sociobiological features our results really are. The relevance of such concern is at least twofold. First, we used ECV as a proxy for some measure of "cognitive reservoir", which ToM could eventually be scaffolded upon. However, ECV also grows with "non-cognitive" brain mass (e.g., cerebellum, basal forebrain, etc…), which is why other measures such as relative neocortex volume have been sometimes preferred. Although the two measures are known to correlate with each other [14,78,79], considering relative neocortex volume instead of ECV may make a difference for, e.g., gorillas, which have a relatively small neocortex given their total brain volume. Second, field estimates of group size in the wild are notoriously debated for orangutans species, which may evolve in so-called "fission-fusion societies" [80]. In our context, this calls for a critical reappraisal of their semi-solitary status (see S2 Text), eventually revising their estimated community size by one order of magnitude. Having said this, it turns out that the conclusion of our analyses does not change if we regress ToM sophistication against relative neocortex volume instead of ECV (neocortex ratio: p = 0.03, group size: p = 0.97), even if we modify orangutans' group size estimate (neocortex ratio: p = 0.04, group size: p = 0.95).

In addition, we acknowledge that other important factors may eventually determine primates' social cognitive skills. Examples include, but are not limited to: flexibility of social hierarchies [81] or dietary constraints on foraging strategies [82]. The issue with considering such sociobiological constraints is twofold. Whether and how they complement or moderate simpler features such as ECV or group size cannot be predicted from first (evolutionary) principles [16]. In fact, this may critically depend on how they are operationally defined. More pragmatically speaking, exploring these dimensions would require testing a huge amount of species in order to compensate for likely statistical correlations between explanatory variables. Taken together, we think it is beyond the scope of the present study to commit to such an exhaustive assessment of the candidate social and biological determinants of animal cognitive skills.

Second, one may challenge our computational definition of ToM, whose least sophisticated form simply cares about others' instrumental reaction to one's actions [47]. Recall that the algorithmic complexity of such "influence learning" scheme lies somewhere between that of *0-ToM* and *1-ToM*. Interestingly, although it is in principle possible to augment the "influence learning" rule with higher-order adjustment terms (cf. Eq 5 in the Methods section), this does not bring any significant behavioural change [49]. This contrasts with *k-ToM* learners, whose depth of recursive beliefs critically determines the expected outcome of social interactions [44]. Note that, in our previous investigation of ToM sophistication in healthy human adults, we found that people mostly behave as either *1-ToM* (estimated frequency = 26%) or *2-ToM* (estimated frequency = 59%) meta-Bayesian agents [49]. We found no strong evidence for such recursive ToM belief update schemes in non-human primates. This implies that meta-Bayesian recursive belief updating schemes may be the hallmark of human social cognition. As we have discussed earlier, the lack of evidence for meta-Bayesian learning in monkeys and apes is in line with the notion of an evolutionary gap between human and non-human minds [39]. But this is not to say that apes lack anything remotely resembling ToM. This is because they behave as if they were adjusting their estimate of others' likely responses to their own actions. Recall that this adjustment depends upon others' covert (cooperative or competitive) intentions. Although it is beyond the grasp of such "influence learning" to realize that others may be using ToM themselves (in contrast to, e.g., *2-ToM*), we argue that it should be seen as a precursor form of ToM in its own right.

In conclusion, although this work does not resolve the debate regarding whether ToM is a uniquely human cognitive skill, it provides an unprecedented computational insight onto the evolutionary roots of social intelligence. In particular, we provide empirical evidence against an orthodox variant of the Machiavellian intelligence hypothesis, which would state that sophisticated ToM evolved mostly as an "on-demand" response to complex societies. Rather, the evolution of sophisticated ToM seems to be mainly determined by neurobiological limiting factors such as the species' "cognitive reservoir". Importantly also, the sophistication of non-human primates' ToM culminates in some form of cognitive precursor of human ToM, or *proto-ToM*. These results are compatible with the idea that ToM may be a byproduct of evolutionary pressure on non-social cognitive skills, which, in conjunction with rigid social norms and/or hierarchies, may otherwise be sufficient to solve most social challenges in most primate species.

## Methods

### Ethics statement

Animals' care and behavioural assessment was performed in accordance with institutional ethical guidelines.

### Experimental methods

The experiments were carried out in four different institutions: the *Institut du Cerveau et de la Moelle épinière* (Paris, France), the *Ménagerie du Jardin des Plantes* (Paris, France), the *St Martin-la-Plaine zoo* (France) and the *Bioparco* (Roma, Italy). Seven primate species were sampled as follows: N = 7 orangutans (*Pongo pygmeus*), N = 6 chimpanzees (*Pan troglodytes)*, N = 5 western gorillas (*Gorilla gorilla*), N = 4 lion-tailed macaques (*Macaca silenus*), N = 5 rhesus macaques (*Macaca mulatta*), N = 9 sooty mangabeys (*Cercocebus atys lunulatus*) and N = 4 ring-tailed lemurs (*Lemurs catta*). This gives an average of about 5.7 ± 1.8 individuals per species. We refer the interested reader to S1 Text for additional information regarding individual characteristics (e.g., sex, age, rearing) these and species' sociobiological features (social group size and ECV).

The protocol consisted in two phases: a habituation/training and an experimental phase, which occurred right before the daily food delivery to keep animals motivated. The food reward was matched to the animal body size (e.g., one or two pieces of dried grapes or papaya) and was kept constant across the entire protocol. The experimenter (a familiar caregiver) always faced the animal in front of the cage (through which the animal could pass their hands or fingers) and positioned his two hands symmetrically (to avoid postural biases). To prevent any olfactory detection of the hiding hand, the caregiver carefully rubbed both hands with the food reward before each test.

The habituation phase was introduced to teach the animal that the reward was hidden in one hand only (before their choice), that a trial begins by the presentation of the caregiver's closed hands, and that it would obtain the content of the hand it would touch or point at. It consisted of two distinct steps. In the first step, the caregiver placed the food reward in one hand and a small stone in the other. Then, he presented both open hands to the animal, such that both contents were clearly visible. The animal received the food only when it touched or clearly pointed uniquely the hand containing the reward. Rewarded side was counterbalanced across trials according to a pseudorandom sequence. This first step was considered successful once the animal reached 10 consecutive correct answers. The second step consisted of a series of three sequences of five trials each: (i) the caregiver first showed both open hands (while attended by the animal) but then closed the non-rewarded hand, (ii) he first showed both open hands and then closed the rewarded hand, and (iii) he first showed both open hands and then closed both hands. In all cases, the individual had to choose the correct hand to obtain the reward. The second step was considered successful once the individual made no error through the entire set of trials (if unsuccessful, the three steps were repeated).

The proper experimental phase began after successful habituation/training, and was grouped into 4x3 = 12 daily sessions of 60 trials each. The order of the three game conditions (*RB*, *0-ToM* or *1-ToM*) were counterbalanced across the 12 sessions, but each game condition was performed by a specific caregiver (counterbalanced across subjects). All sessions were video-recorded. If a daily session was interrupted for more than 10 minutes (because of, e.g., frustration or attentional distraction), the session was terminated and possibly restarted on another day. Only sessions longer than 20 trials were included in the final analysis. At each trial, the caregiver presented his two hands closed after having hidden the food reward and the stone out of the animal's sight. If the animal chose the correct hand, he was allowed to take and eat the food reward. Otherwise, the caregiver acted as if he was eating the food while exaggerating chewing, vocalizing pleasure and staring at the animal. The reward location was instructed by the algorithm corresponding to the game condition (*RB*, *0-ToM* or *1-ToM*). This required the presence of a co-experimenter who entered the individual's response into a laptop computer at each trial, enabling the model to compute on-line the reward location at the next trial.

### Computational modelling of learning styles

In this section, we give a brief overview of the set of candidate learning models, with a particular emphasis on *k-ToM* models (because these are also used as on-line algorithms during the experimental phase). We will consider repeated dyadic (two-players) games, in which only two actions are available for each player (the animal and the caregiver). Hereafter, the action of a given agent (resp., his opponent) is denoted by *a*^*self*^ (resp., *a*^*op*^). A game is defined in terms of its payoff table, whose entries are the player-specific utility *U*(*a*^*self*^,*a*^*op*^) of any combination of players' actions at each trial. In particular, competitive (resp., cooperative) social interactions simply reduce to anti-symmetric (resp. symmetric) players’ payoff tables (see tables S3 and S4 in S1 Text).

By convention, actions *a*^*op*^ and *a*^*self*^ take binary values encoding the first (*a* = 1) and the second (*a* = 0) available options. According to Bayesian decision theory, agents aim at maximising expected payoff *V* = *E*[*U*(*a*^*self*^,*a*^*op*^)], where the expectation is defined in relation to the agent's uncertain predictions about his opponent's next move. This implies that the form of the decision policy is the same for all agents, irrespective of their ToM sophistication. Here, we consider that choices may exhibit small deviations from the rational decision rule, i.e. we assume agents employ the so-called "softmax" probabilistic policy:

$$P\left(a^{self}=1\right)=\frac{1}{1+\exp\left(-\frac{\Delta V}{\beta }\right)}$$ (1)

where *P*(*a*^*self*^ = 1) is the probability that the agent chooses the action *a*^*self*^ = 1, Δ*V* is the expected payoff difference (between actions *a*^*self*^ = 1 and *a*^*self*^ = 0), and *β* is the so-called behavioural "temperature" (which controls the magnitude of deviations from rationality). The sigmoidal form of Eq 1 simply says that the probability of choosing the action *a*^*self*^ = 1 increases with the expected payoff difference Δ*V*, which is given by:

$$\Delta V=p_{}^{op}\left(U\left(1,1\right)-U\left(0,1\right)\right)+\left(1-p_{}^{op}\right)\left(U\left(1,0\right)-U\left(0,0\right)\right)$$ (2)

where *p*^*op*^ is the probability that the opponent will choose the action *a*^*op*^ = 1. This prediction is critical, in that it provides the agent with prospective action values. For example, if one believes that the opponent is likely to pick action *a*^*op*^ = 1 (i.e. if *p*^*op*^ ≈ 1), then the expected payoff reduces to Δ*V* = *U*(1,1)−*U*(0,1), which directly determine the incentive towards choosing either *a*^*self*^ = 1 or *a*^*self*^ = 0. In our context, animals are rewarded for choosing the hand in which the caregiver has hidden the food reward, which is simply written as: *U*(1,1)−*U*(0,1) = *U*(0,0)−*U*(1,0) = 1 ⇒ Δ*V* = 2*p*^*op*^ −1.

Let us first disclose the intuition behind *k-ToM* models, which essentially differ in how they estimate *p*^*op*^. We refer the interested reader to S1 Text for a more detailed mathematical description. In brief, the repeated observation of his opponent's behaviour (*a*^*op*^) gives the agent the opportunity to learn his opponent's behavioural tendency *p*^*op*^. Theory of Mind comes into play when agents consider that *p*^*op*^ is driven by the opponent's hidden beliefs and desires. More precisely, *k-ToM* agents consider that the opponent is himself a Bayesian agent, whose decision policy *p*^*op*^ = *P*(*a*^*op*^ = 1) is formally similar to Eq 1. In this situation, *k-ToM* agents have to track their opponent's prediction *p*^*self*^ about their own actions. In line with [42], this meta-Bayesian inference is based upon recursive belief updating ("I believe that you believe that I believe…"). The recursion depth *k* induces distinct ToM sophistication levels, which differ in how they update their subjective prediction *p*^*op*^, hence *k-ToM*. More formally, *k-ToM* learning agents are defined recursively, starting with *0-ToM*.

By convention, a *0-ToM* agent does not attribute mental states to his opponent, but rather tracks his overt behavioural tendency without mentalizing. More precisely, *0-ToM* agents simply assume that their opponents choose the action *a*^*op*^ = 1 with probability *p*^*op*^ = *s*(*x*_*t*_), where the log-odds *x*_*t*_ varies across trials *t* with a certain volatility *σ*^0^ (and *s* is the sigmoid function). Observing his opponent's choices gives *0-ToM* information about the hidden state *x*, which can be updated trial after trial using Bayes rule, as follows:

$$\begin{array}{c} \mu_{t}^{0}\approx \mu_{t-1}^{0}+\Sigma_{t}^{0}\left(a_{t}^{op}-s\left(\mu_{t-1}^{0}\right)\right)\\ \Sigma_{t}^{0}\approx \frac{1}{\frac{1}{\Sigma_{t-1}^{0}+\sigma^{0}}+s\left(\mu_{t-1}^{0}\right)\left(1-s\left(\mu_{t-1}^{0}\right)\right)} \end{array}$$ (3)

where $\mu_{t}^{0}$ (resp. $\Sigma_{t}^{0}$) is the approximate mean (resp. variance) of *0-ToM*'s posterior distribution $p\left(x_{t}^{0}|a_{1:t}^{op}\right)$. Inserting ${\hat{p}}_{t+1}^{op}=E\left[s\left(x_{t+1}\right)|a_{1:t}^{op}\right]$ into Eq 1 now yields *0-ToM*'s decision rule. Here, the effective learning rate is the subjective uncertainty ∑^0^, which is controlled by the volatility *σ*^0^. At the limit *σ*^0^ → 0, Eq 3 converges towards the (stationary) opponent's choice frequency and *0-ToM* essentially reproduce "fictitious play" strategies [50,83].

*0-ToM*'s learning rule is the starting point for a *1-ToM* agent, who considers that she is facing a *0-ToM* agent. This means that *1-ToM* has to predict *0-ToM*'s next move, given his beliefs and the choices' payoffs. The issue here is that *0-ToM*'s parameters (volatility *σ*^0^ and exploration temperature *β*) are unknown to *1-ToM* and have to be learned, through their non-trivial effect on *0-ToM'*s choices. At trial *t* + 1, a *1-ToM* agent predicts that *0-ToM* will chose the action *a*^*op*^ = 1 with probability $p_{t+1}^{op,0}=s\circ v^{0}\left(x_{t}^{0},a_{\to t}^{}\right)$, where the hidden states $x_{t}^{0}$ lumps *σ*^0^ and *β* together and the mapping *v*^0^ is derived from inserting *0-*ToM's learning rule (Eq 3) into Eqs 1 and 2. Similarly to *0-ToM* agents, *1-ToM* assumes that the hidden states $x_{t}^{0}$ vary across trials with a certain volatility *σ*^1^, which yields a meta-Bayesian learning rule similar in form to *0-ToM*'s, but relying on first-order meta-beliefs (i.e. beliefs about beliefs). In brief, *1-ToM* eventually learns how her (*0-ToM*) opponent learns about herself, and acts accordingly (cf. Eqs 1 and 2).

*1-ToM* agents are well equipped to deal with situations of observational learning. However, when it comes to reciprocal social interactions, one may benefit from considering that others are also using ToM. This calls for learning styles that rely upon higher-order meta-beliefs. By construction, *k-ToM* agents (*k* ≥ 2) consider that their opponent is a *κ-ToM* agent with a lower ToM sophistication level (i.e.: *κ* < *k*). Importantly, the sophistication level *κ* of *k-ToM*'s opponent has to be learned, in addition to the hidden states *x*^*κ*^ that control the opponent's learning and decision making. The difficulty for a *k-ToM* agent is that she needs to consider different scenarios: each of her opponent's possible sophistication level *κ* yields a specific probability $p_{t+1}^{op,\kappa }=s\circ v^{\kappa }\left(x_{t}^{\kappa },a_{\to t}^{}\right)$ that she will choose action *a*^*op*^ = 1. The ensuing meta-Bayesian learning rule entails updating *k-ToM*'s uncertain belief about her opponent's sophistication level *κ* and hidden states *x*^*κ*^:

$$\begin{array}{c} \lambda_{t}^{k,\kappa }\approx {\left[\frac{\lambda_{t-1}^{k,\kappa }\;p_{t}^{op,\kappa }}{\sum_{\kappa '<k}\lambda_{t-1}^{k,\kappa '}\;p_{t}^{op,\kappa '}}\right]}^{a_{t}^{op}}{\left[\frac{\lambda_{t-1}^{k,\kappa }\;\left(1-p_{t}^{op,\kappa }\right)}{\sum_{\kappa '<k}\lambda_{t-1}^{k,\kappa '}\;\left(1-p_{t}^{op,\kappa '}\right)}\right]}^{1-a_{t}^{op}}\\ \mu_{t}^{k,\kappa }\approx \mu_{t-1}^{k,\kappa }+\lambda_{t}^{\kappa }\;\Sigma_{t}^{k,\kappa }\;W_{t-1}^{\kappa }\left(a_{t}^{op}-s\circ v^{\kappa }\;\left(\mu_{t-1}^{k,\kappa }\right)\right)\\ \sum_{t}^{k,\kappa }\approx {\left[{\left(\sum_{t-1}^{k,\kappa }+\sigma^{k}\right)}^{-1}+s'\circ v^{\kappa }\left(\mu_{t-1}^{k,\kappa }\right)\;\lambda_{t}^{\kappa }\;W_{t-1}^{\kappa }{}^{T}W_{t-1}^{\kappa }\right]}^{-1} \end{array}$$ (4)

where $\lambda_{t}^{k,\kappa }$ is *k-ToM*'s posterior probability that her opponent is *κ*-*ToM*, and *W*^*κ*^ is the gradient of *v*^*κ*^ with respect to the hidden states *x*^*κ*^. Note that although the dimensionality of *k-ToM*'s beliefs increases with *k*, *k-ToM* models do not differ in terms of the number of their free parameters. More precisely, *k-ToM*’s learning and decision rules are entirely specified by their prior volatility *σ*^*k*^ and behavioural temperature *β*. Finally, the only difference between "competitive" and "cooperative" *k-ToM* learners lies in the specification of the utility table *U*(*a*^*self*^, *a*^*op*^). Although it is held constant across trials, it can induce profound changes in the effective learning style of *k-ToM* agents [44,49]. We refer the interested reader to the S1 Text for mathematical details regarding *k-ToM* learning models.

Critically, only *k-ToM* agents with *k*≥1 are learning about others' covert mental states (by updating meta-beliefs). This would suggest a clear sophistication cut-off for discriminating ToM and no-ToM learning styles. But in fact, we will also consider a hybrid (non Bayesian) model that somehow lies in between *0-ToM* and *1-ToM*, and still qualifies for ToM. We refer the interested reader to [47] for a mathematical derivation of the "influence learning" model. In brief, it is essentially a *0-ToM* learner that heuristically adjusts his learning rule to account for how her own actions influence her opponent’s strategy:

$$p_{t+1}^{op}=p_{t}^{op}+\eta \underset{\mathrm{prediction}\;\mathrm{error}}{\underbrace{\left(a_{t}^{op}-p_{t}^{op}\right)}}-\lambda \underset{“\mathrm{influence}”\;\mathrm{adjustment}\;\mathrm{term}}{\underbrace{p_{t}^{op}\left(1-p_{t}^{op}\right)\left(2a_{t}^{self}+\left(2I^{comp}-1\right)\beta s^{-1}\left(p_{t}^{op}\right)+I^{comp}\right)}}$$ (5)

where *η* (resp. *λ*) controls the relative weight of its prediction error (resp. the “influence” adjustment term), and *I*^*comp*^ is a binary indicator variable for the type of social interaction (competition: *I*^*comp*^ = 1, cooperation: *I*^*comp*^ = 0). In contrast to *1-ToM*, this learning rule bypasses any form of recursive belief update. However, *Inf* explicitly depends upon the other player's covert (competitive or cooperative) intention, which is beyond the grasp of *0-ToM*. In analogy with *k-ToM* models, it is in principle possible to augment Eq 5 with higher-order adjustment terms. This, however, has little effect on the way the algorithm learns [49]. In addition, numerical simulations show that, in a competitive game setting, *Inf* wins over *0-ToM* but loses against *1-ToM*. This is why, altogether, we think of "influence learning" as some form of proto-ToM.

With the exception of *0-ToM*, we so far only described sophisticated learning models that are capable of (artificial) ToM. But even *0-ToM* can be considered too sophisticated for some primate species. In the aim of assessing the evidence for ToM sophistication (from primates' choice sequences), we thus have to benchmark the above models against simpler learning styles that involve even fewer cognitive resources. We will describe three of these "unsophisticated" learning models below.

First, animals may learn by trial and error, eventually reinforcing the actions that led to a reward. Such learning style is the essence of classical conditioning, which is typically modelled using reinforcement learning or *RL* [84]. In this perspective, animals would directly learn the value of alternative actions, which bypasses Eq 2. More precisely, an *RL* agent would update the value of the chosen option in proportion to the reward prediction error, as follows:

$$\left\{ \begin{array}{l} V_{t+1}^{i}=V_{t}^{i}+\alpha \left(R_{t}-V_{t}^{i}\right)\;\mathrm{if}\;\mathrm{action}\;a_{t}^{self}=i\;\mathrm{was}\;\mathrm{chosen}\\ V_{t+1}^{i}=V_{t}^{i}\;\mathrm{otherwise} \end{array}\right.$$ (6)

where $R_{t}=U\left(a_{t}^{self},a_{t}^{op}\right)$ is the last reward outcome and *α* is the (unknown) learning rate. At the time of choice, animals simply tend to pick the most valuable option (cf. Eq 1).

Second, an even simpler way of adapting one's behaviour in operant contexts such as this one is to repeat one's last choice if it was successful and alternate otherwise. This can be modeled by the following update in action values:

$$\left\{ \begin{array}{l} V_{t+1}^{i}=R_{t}\;\mathrm{action}\;a_{t}^{self}=i\;\mathrm{was}\;\mathrm{chosen}\\ V_{t+1}^{i}=-R_{t}\;\mathrm{otherwise} \end{array}\right.$$ (7)

This strategy is called win-stay/lose-switch (*WS*), and is almost identical to the above *RL* model when the learning rate is *α* = 1. Despite its simplicity, *WS* can be shown to have remarkable adaptive properties [85].

Last, the agent may simply act randomly, which can be modeled by fixing the value difference to zero (Δ*V* = 0). Although embarrassingly simple, this probabilistic policy eventually prevents one's opponent from controlling one's expected earnings. It thus minimizes the risk of being exploited at the cost of providing chance-level expected earnings. It is the so-called "Nash equilibrium" of our "hide and seek" game [86]. Since we augment this chance model with a potential bias for one of the two alternative options (as all the above learning models), we refer to it as *biased Nash* or *BN*.

### Statistical data analyses

Our statistical data analysis proceeds in three steps of increasing specificity, namely: multiple regression of behavioural performances, Volterra decompositions of trial-by-trial choice sequences and Bayesian model comparison. All statistical analyses were performed using the VBA toolbox [87].

First, let us summarize our random-effect analysis of performance. As a preliminary stage, we regressed out the effect of session repetition and time elapsed since the last experimental session from measured individual performances. We then reported the adjusted individual performance scores per opponent at the group level. We regressed performance against the effect of species, opponent (conditions *RB*, *0-ToM* and *1-ToM*), and their interaction. In addition to subject-specific intercepts, we also included the interactions of the opponent effect with age (normalized by species-specific life time expectancy in the wild), sex and rearing (wild vs captivity). In turn, statistical tests for effects of species and opponent assess significance above and beyond these potential inter-individual differences. The specific effects of ECV and group size were tested using weighted linear contrasts.

Second, we performed Volterra decompositions of trial-by-trial choice sequences using session-specific Bayesian logistic regressions, as follows:

$$\begin{array}{c} p\left(a_{}^{self}|\omega \right)=\prod_{t}q_{t}{\left(\omega \right)}^{a_{t}^{self}}{\left(1-q_{t}\left(\omega \right)\right)}^{1-a_{t}^{self}}\\ q_{t}\left(\omega \right)=s\left(\omega^{0}+\sum_{\tau }\omega_{\tau }^{op}\left(2a_{t-\tau }^{op}-1\right)+\sum_{\tau }\omega_{\tau }^{self}\left(2a_{t-\tau }^{self}-1\right)\right) \end{array}$$ (8)

where $q_{t}\left(\omega \right)=p\left(a_{t}^{self}=1|\omega \right)$ is the probability that the agent chooses the first option at trial *t*, *τ* is some arbitrary time lag and *ω* is the so-called Volterra kernel (*ω*^0^ is a potential bias for one of the alternative options). Volterra kernels *ω*^*op*^ (resp. *ω*^s*elf*^) capture the impact of lagged opponent's (resp. own) actions *a*^*op*^ (resp. *a*^*self*^) onto primates’ choice probability. For the sake of efficiency, we further reduce the Volterra kernels to parameterized exponential mappings, i.e.: *ω*_*τ*_ = *A*exp(−*λτ*), where *A* (resp. *λ*) is the kernel's magnitude (resp. temporal decay). For each individual and each session, we fit the resulting model and report the kernels' magnitudes *A*^*op*^ and *A*^*self*^ at the group level. The ensuing random-effect analyses are identical to the above performance scores.

Third, we performed statistical (Bayesian) model comparisons. For each subject, we fitted the above ten learning models on trial-by-trial action sequences using a variational-Laplace approach [88,89]. Different sessions of the same opponent condition were pooled together, allowing us to constrain the model parameters to be identical across sessions (but not across opponents). Eventually, we obtained 10x35 = 350 model evidences (10 models and 35 individuals; the 3 opponent conditions were lumped together for model inversions). These model evidences were partitioned into ToM (*1-ToM*, *2-ToM* and *Inf*) and no-ToM (all other models) families, to obtain within-subject posterior probabilities *pToM* of exhibiting a ToM-compatible learning style. These scores were then averaged across individuals within species to yield the variable *pToM*, for further analyses (see Fig 5). In addition, we performed a group-level random-effect Bayesian model comparison [54,90]. In particular, this analysis enabled us to estimate the frequency profiles of learning models within species with high versus low ECV. We refer the interested reader to S1 Text for additional statistical details regarding the Bayesian model comparison.

## Supporting information

S1 Text This document contains additional details regarding methods (species' feature variables, *k-ToM* learning model, control task, Bayesian model comparison, phylogenic analyses) and additional results (performance, Volterra analyses, fit accuracy of learning models, confusion analyses for model comparison). (DOCX)

S2 Text This document contains a table summarizing all reported species' group size data as well as the list of all corresponding source references. (DOCX)
