# Correction: Reading wild minds: A computational assay of Theory of Mind sophistication across seven primate species

**DOI:** 10.1371/journal.pcbi.1005917

**Published:** 2017-12-20

**Authors:** The PLOS Computational Biology Staff

The affiliations of Christelle Hano, Giulia Bardino and Michel Saint Jalme are listed incorrectly. The correct affiliations are as follows:

Christelle Hano

**Affiliation:** Museum National d'Histoire Naturelle, UMR 7204, Ménagerie du Jardin des Plantes, Paris, France.

Giulia Bardino

**Affiliation:** Museum National d'Histoire Naturelle, UMR 7206, Universita La Sapienza, Rome, Italy

Michel Saint Jalme

**Affiliation:** Museum National d'Histoire Naturelle, UMR 7204, Ménagerie du Jardin des Plantes, Paris, France.

There is also an error in the author contributions. The listed roles for Shelly Masi should read as follows:

Shelly Masi

**Roles:** Conceptualization, Formal analysis, Funding acquisition, Investigation, Resources, Supervision, Writing–original draft, Writing–review & editing
