# Supplementary material for: Reading wild minds: A computational assay of Theory of Mind sophistication across seven primate species
Source: PLoS Comput Biol. 2017 Nov 7;13(11):e1005833. doi: 10.1371/journal.pcbi.1005833 (PMC5693450; doi:10.1371/journal.pcbi.1005833)
Supplement: S1 Text — (DOCX) [file pcbi.1005833.s001.docx]

***Additional Methods***

***Species***

Table S1 below summarizes the sociobiological features of the seven primate species investigated in this study (as well as *Homo Sapiens*, for comparison purposes).

| **Species** | **Common name** | **Group size** | **ECV (cc)** | **Neocortex ratio** |
| --- | --- | --- | --- | --- |
| *Lemur catta* | lemur | 15.0 | 22.9 | 1.18 |
| *Macaca silenus* | lion-tailed macaque | 30.7 | 80.0 | 2.36 |
| *Macaca mulatta* | rhesus macaque | 34.8 | 88.3 | 2.60 |
| *Cercocebus* | sootey mangabey | 54.1 | 94.7 | 2.38 |
| *Gorilla gorilla* | western gorilla | 8.5 | 501.5 | 2.65 |
| *Pongo* | orangutan | 1.7 | 249.3 | 3.17 |
| *Pan troglodyte* | chimpanzee | 43.1 | 357.6 | 3.22 |
| *Homo sapiens* | human | 150 | 1195.0 | 4.10 |

Table S1. Species-specific sociobiological features**.** ECV (endocranial volume) was obtained from a published database (Isler et al., 2008). Here, we simply report the average group size across published field studies. The corresponding list of source references is given in the Supplementary Text S2. Neocortex ratios are taken from (Dunbar, 1992), which extracted them from (Stephan et al., 1981).

In the main text, we use ECV as a measure of species' "cognitive reservoir", and group size as a proxy for the complexity of species' societies. This is supported by meta-analytic studies showing that overall brain size is the best predictor of general cognitive ability across non-human primates (Deaner et al., 2007). Nevertheless, it may be argued that our approach is plagued with two sorts of limitations.

First, our measures may be confounded by artefactual noise. For example, our set of species can be partitioned into grades of distinct socio-cognitive complexity (e.g. apes versus other simians and prosimians). This can be accounted for by including grade offsets (van Schaik and Burkart, 2011) when regressing species' feature variables (ECV, group size, neocortex ratio) against each other. In addition, our reported group sizes does not account for fission-fusion types of societes (e.g., orangutans' group size is derived under the assumption that they are semi-solitary). Solving this issue simply consists in replacing orangutans' group size by the reported size of the wider fission-fusion community (van Schaik, 1999), namely: 11. Figure S1 below summarizes the ensuing statistical relationship between ECV and group size.


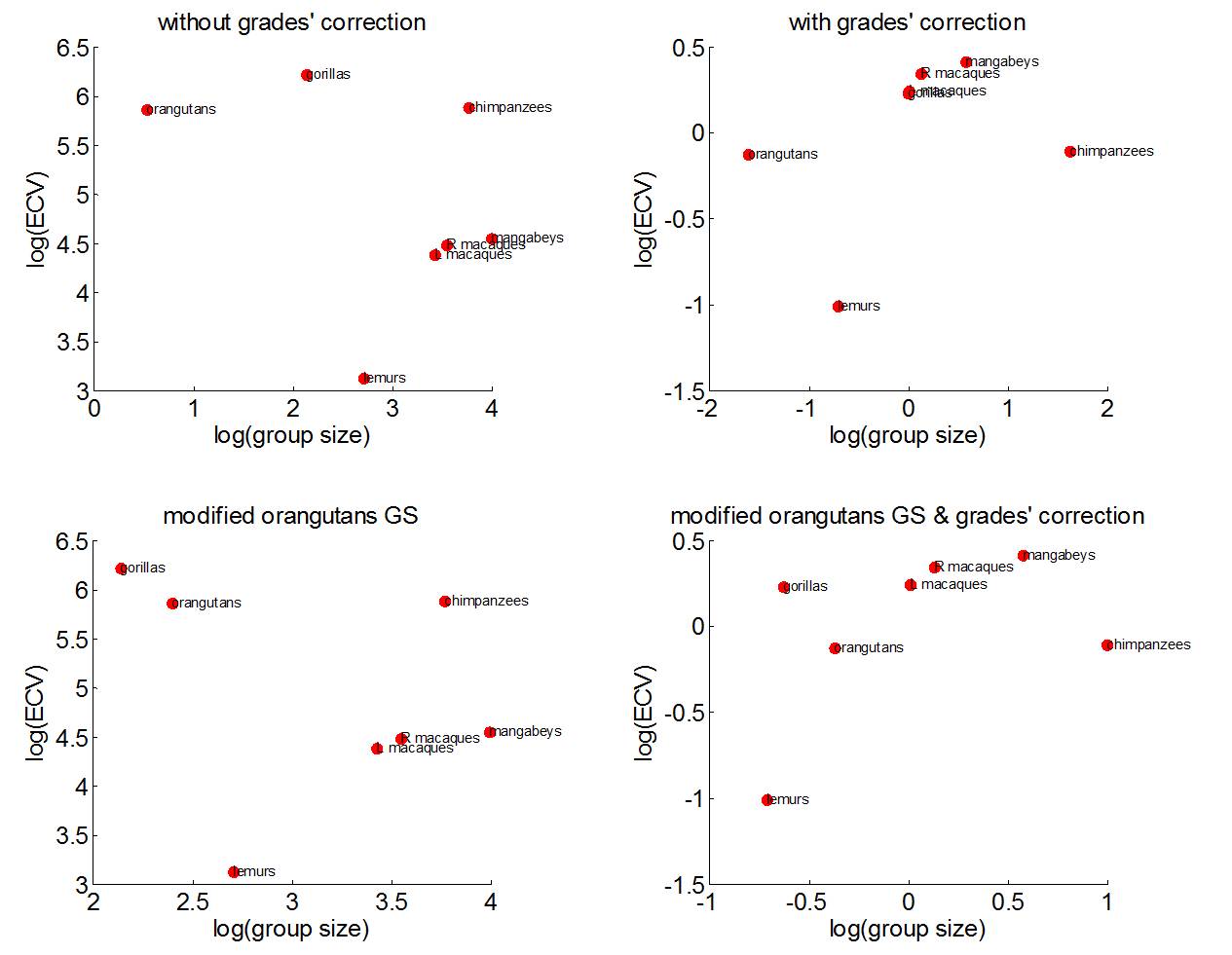


Figure S1: sociobiological features of tested non-human primates species. On each graph, log social group size (x-axis) is plotted as a function of log ECV (y-axis). Top-left: without any correction (raw data, as in Fig 1 of the main text), Top-right: with grade correction, Bottom-left: with modified orangutans' group size, Bottom-right: with grade's correction and modified orangutans' group size.

Although one can see that the correlation between ECV and group size visually improves as corrections are included, it turns out that no correlation is statistically significant (without any correction: r=-0.37, p=0.41 ; with grade correction: r=0.63, p=0.49 ; with modified orangutans' group size: r=-0.27, p=0.56 ; with both corrections: r=0.61, p=0.08).

Second, ECV also grows with "non-cognitive" brain mass (e.g., cerebellum, basal forebrain, etc...). This criticism calls for an even more radical correction, which, in our case, consists in replacing ECV with relative neocortex volume (i.e. neocortex ratio). The other two corrections can then be applied (to neocortex ratio and group size), as is summarized in Figure S2 below.


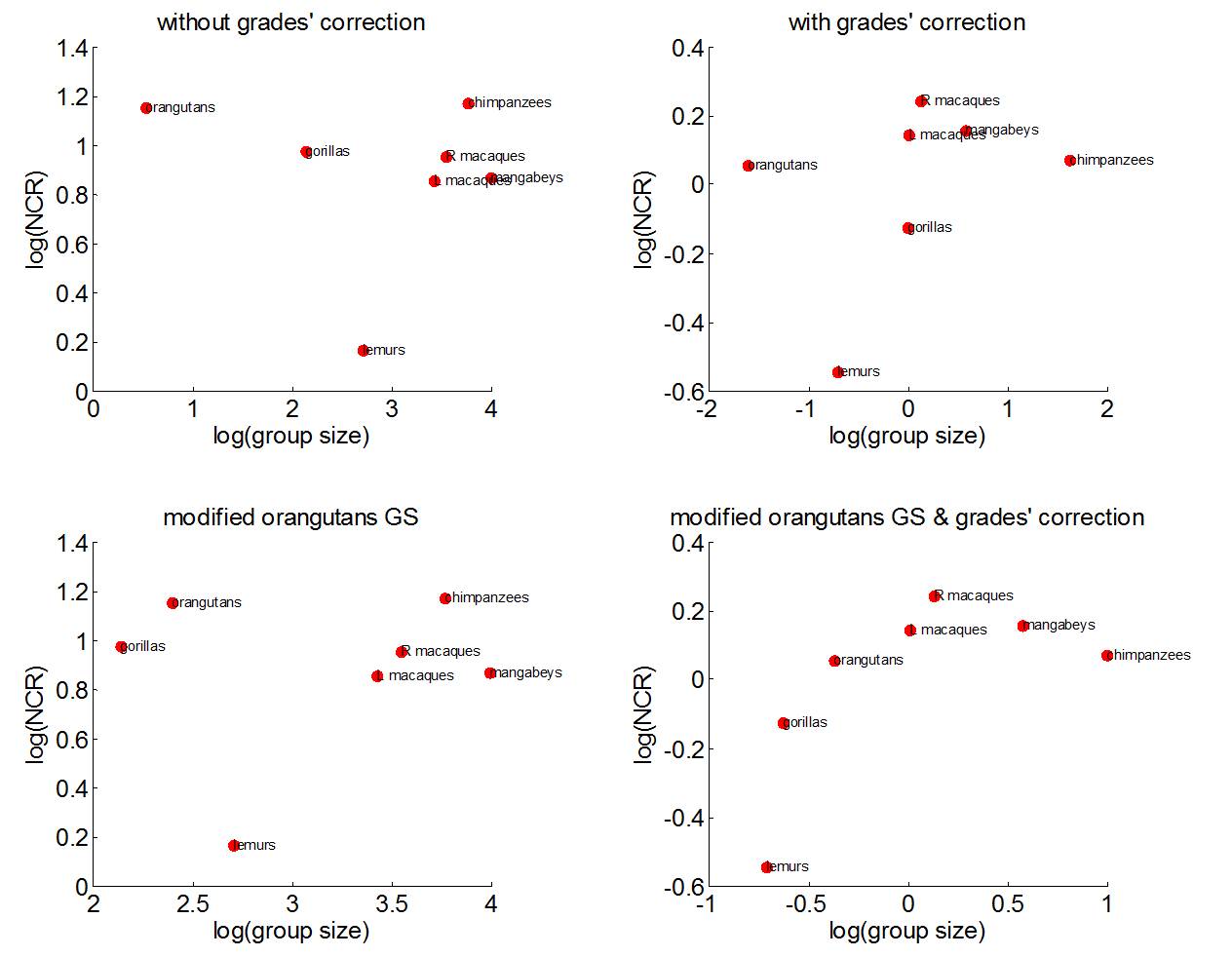


Figure S2: sociobiological features of tested non-human primates species. On each graph, log social group size (x-axis) is plotted as a function of log neocortex ratio (y-axis). Same format as Fig S1.

Here again, no correlation turns out be statistically significant (without any correction: r=-0.12, p=0.78 ; with grade correction: r=0.62, p=0.51 ; with modified orangutans' group size: r=0.14, p=0.76 ; with both corrections: r=0.73, p=0.17). In brief, absolute brain volume (ECV) and relative neocortex volume (NCR) yield similar results, which is not surprising, given that the latter has been shown to behave as an exponential function of the latter across primate species (Finlay and Darlington, 1995).

The interim conclusion at this point is that none of these corrections actually recovers the expected statistical relationship between brain volume and group size (Dunbar, 1992, 1995). For the sake of simplicity, we therefore decided to work with uncorrected ECV and group size variables. We comment on this and related issues in the Discussion Section of the main text.

***Subjects***

Table S2 below provides additional information on the tested individuals. These variables were considered as confounds for our analyses, and included as nuisance variables in the GLM analyses.

| **Individuals** | **Species common name** | **Sex** | **Age/lifespan** | **Rearing** |
| --- | --- | --- | --- | --- |
| Cloug | sootey mangabey | M | 0.92 | Parent |
| Cumba* | sootey mangabey | F | 0.08 | hand |
| Loango | sootey mangabey | M | 0.08 | hand |
| Luca | sootey mangabey | M | 0.24 | Parent |
| Quorum | sootey mangabey | M | 0.12 | Hand |
| Arturo | sootey mangabey | M | 0.16 | Hand |
| Agata | sootey mangabey | F | 0.00 | Hand |
| Ashante | sootey mangabey | F | 0.60 | Parent |
| Hindie | Lion-tailed macaque | M | 0.35 | Parent |
| Effrat | Lion-tailed macaque | F | 0.45 | Parent |
| Ella | Lion-tailed macaque | F | 0.20 | Parent |
| Aya | Lion-tailed macaque | F | 0.35 | Parent |
| Joey* | Orangutan | M | 0.33 | Parent |
| Tamu | Orangutan | F | 0.13 | Parent |
| Théodora | Orangutan | F | 0.44 | Parent |
| Nénette | Orangutan | F | 0.78 | Parent |
| Petronilla | Orangutan | F | 0.80 | Hand |
| Zoe | Orangutan | F | 0.53 | Hand |
| Martina** | Orangutan | F | 0.45 | Hand |
| Aliocha | Rhesus macaque | M | 0.28 | Parent |
| Dracula | Rhesus macaque | M | 0.24 | Parent |
| Bob | Rhesus macaque | M | 0.24 | Parent |
| Rose | Rhesus macaque | F | 0.64 | Parent |
| Chanel | Rhesus macaque | F | 0.20 | Parent |
| Edy | Chimpanzee | F | 0.51 | Hand |
| Bingo | Chimpanzee | M | 0.56 | Hand |
| Doly | Chimpanzee | F | 0.74 | Hand |
| Bambou | Chimpanzee | M | 0.42 | Hand |
| Clementine | Chimpanzee | F | 0.67 | Parent |
| Kuma | Chimpanzee | M | 0.23 | Parent |
| Pamela | Western gorilla | F | 1.06 | Parent |
| Hyasmina | Western gorilla | F | 1.03 | Parent |
| Digit | Western gorilla | F | 0.46 | Hand |
| Likalè | Western gorilla | M | 0.37 | Parent |
| Euro | Western gorilla | M | 0.43 | Parent |
| Toto | Ring-tailed Lemur | M | 0.06 | Parent |
| Tootsie | Ring-tailed Lemur | F | 0.11 | Parent |
| Nando* | Ring-tailed Lemur | M | 0.17 | Parent |
| Nene* | Ring-tailed Lemur | F | 0.17 | Parent |

Table S2: Inter-individual variables. The age is normalized by lifespan of the species in the wild. Regarding rearing, “hand” means that the corresponding individual was raised by a human. Individuals who were not tested in the control task (see below) are marked with an asterisk. One individual showed signs of a rare and severe genetic disease equivalent to human Down syndrome (see, e.g., Hirata et al., 2017). She is marked with a double asterisk.

Note that some individuals never succeeded the habituation phase, for lack of interest (six chimpanzees), anxiety of being isolated from the rest of the group (two mangabeys) or apparent failure of understanding (three rhesus macaques). These individuals were not included in the testing phase of the experiment. In addition, habituation was faster in apes than in monkeys who did not understood easily that they could have access to the reward even though the hand were closed.

***k-ToM model***

In this section, we expose the key steps in the derivation of the *k-ToM* model in the context of repeated two-player games. We used this model both to generate the choices of the artificial opponents during the experiment, and in the analysis of individuals’ trial-by-trial choice sequences. First, recall that a game is defined in terms of a utility table $U\left(a^{self},a^{op}\right)$, which yields the payoff one gets when making decision $a^{self}$ while the other player chooses $a^{op}$. Incentives can be arbitrarily chosen to capture different forms of social exchanges or transactions (see below). By convention, actions $a^{op}$ and $a^{self}$ take binary values encoding the first ($a=1$) and the second ($a=0$) available options. According to Bayesian decision theory, agents aim at maximising expected payoff $V=E\left[U\left(a^{self},a^{op}\right)\right]$, where the expectation is defined in relation to the agent's uncertain predictions about his opponent's next move. In this work, we consider that choices may exhibit small deviations from the optimal decision rule, i.e. we assume that agents employ the so-called "softmax" probabilistic policy (see equations 1-2 in the main text).

We define *k-ToM* agents in terms of the way they learn from their opponent's behaviour, starting with *0-ToM*. By convention, a *0-ToM* agent does not attribute mental states to his opponent. More precisely, *0-ToM* agents simply assume that their opponents choose the action $a^{op}=1$ with probability $p^{op}=s\left(x_{t}^{0}\right)$, where the log-odds $x_{t}^{0}$ varies across trials $t$ with a certain volatility $\sigma^{0}$ (and $s$ is the sigmoid function). Observing his opponent's choices gives *0-ToM* information about the hidden state $x^{0}$, which can be updated trial after trial using the following Bayes-optimal probabilistic scheme:

$q\left(x_{t+1}^{0}\right)\propto p\left(a_{t+1}^{0}\left|x_{t+1}^{0}\right.\right)\int q\left(x_{t}^{0}\right)p\left(x_{t+1}^{0}\left|x_{t}^{0}\right.\right)dx_{t}^{0}\begin{matrix} \\ \end{matrix}$ (S1)

where $p\left(x_{t+1}^{0}\left|x_{t}^{0}\right.\right)$ encodes *0-ToM*’s prior belief on the volatility of the log-odds, and $q\left(x_{t}^{0}\right)\equiv p\left(x_{t}^{0}\left|a_{1:t}^{op}\right.\right)$ is his posterior belief about the log-odds $x_{t}^{0}$ at trial $t$, having observed his opponent's behaviour $a^{op}$ up to trial $t$. Under these premises, one can derive *0-ToM*'s learning rule, in terms of the change in his prediction about his opponent's next move:

$\begin{matrix} \hat{p}_{t+1}^{op}\approx s\left(\mu_{t}^{0}/\sqrt{1+\left(\Sigma_{t}^{0}+\sigma^{0}\right)3/\pi^{2}}\right) \\ \mu_{t}^{0}\approx\mu_{t-1}^{0}+\Sigma_{t}^{0}\left(a_{t}^{op}-s\left(\mu_{t-1}^{0}\right)\right) \\ \Sigma_{t}^{0}\approx\frac{1}{\frac{1}{\Sigma_{t-1}^{0}+\sigma^{0}}+s\left(\mu_{t-1}^{0}\right)\left(1-s\left(\mu_{t-1}^{0}\right)\right)} \end{matrix}$ (S2)

where $\mu_{t}^{0}$ (resp. $\Sigma_{t}^{0}$) is the approximate mean (resp. variance) of *0-ToM*'s posterior distribution $q\left(x^{0}\right)$. In other words, $\mu_{t}^{0}$ is *0-ToM*'s estimate of the log-odds at trial $t$, and $\Sigma_{t}^{0}$ is her subjective uncertainty about it. Inserting $\hat{p}_{t+1}^{op}=E\left[s\left(x_{t+1}\right)\left|a_{1:t}^{op}\right.\right]$ into Equation 1 now yields *0-ToM*'s decision rule. Note that the term $a_{t}^{op}-s\left(\mu_{t-1}^{0}\right)$ can be thought of as a prediction error, whose impact on learning accounts for changes in the subjective uncertainty $\Sigma^{0}$. Here, the effective learning rate is controlled by the volatility $\sigma^{0}$. At the limit $\sigma^{0}\to0$, Equation S2 converges towards the (stationary) opponent's choice frequency and *0-ToM* essentially reproduce "fictitious play" strategies (Berger, 2007).

*0-ToM*’s learning rule is the starting point for a *1-ToM* agent, who has to predict *0-ToM*'s next move, given his beliefs and the choices' payoffs. The issue here is that *0-ToM*'s priors (as well as his exploration temperature) are unknown to *1-ToM* and have to be learned, through their non-trivial effect on *0-ToM'*s choices. More precisely, *1-ToM* agents assume that *0-ToM* chooses the action $a^{op}=1$ with probability $p^{op}=s\circ v^{1}\left(x^{1}\right)$, where the hidden states $x^{1}$ lumps $\sigma^{0}$ and $\beta$ together and the mapping $v^{1}$ is derived from inserting Equation S2 into the softmax policy (cf. Equation 1 of the main text):

$\begin{matrix} P\left(a_{t}^{op}=1\right)=s\circ v^{1}\left(x_{t}^{1}\right) \\ v^{1}\left(x_{t}^{1}\right)=\frac{p_{t}^{self}\Delta U_{t}^{1}+\left(1-p_{t}^{self}\right) \Delta U_{t}^{0}}{\beta_{t}} \\ p_{t}^{self}=s\left(\mu_{t-1}^{0}/\sqrt{1+\left(\Sigma_{t-1}^{0}+\sigma_{t}^{0}\right)3/\pi^{2}}\right) \end{matrix}$ (S3)

where $\Delta U_{t}^{i}=U\left(a^{op}=1,a^{self}=i\right)-U\left(a^{op}=0,a^{self}=i\right)$ is the net incitation of *1-ToM*'s opponent to pick the first option if *1-ToM* chooses option $a^{self}=i$. Here, *1-ToM*'s estimate of $p^{self}$ is effectively a second-order belief, i.e. *1-ToM*'s bet about her opponent's prediction about her own next move. Similarly to *0-ToM* agents, *1-ToM* assumes that the hidden states $x_{t}^{1}$ vary across trials with a certain volatility $\sigma^{1}$, which yields a meta-Bayesian learning rule similar in form to Equation S2 (see Equation S4 below). In brief, *1-ToM* eventually learns how her (*0-ToM*) opponent learns about herself, and acts accordingly. Note that, similarly to our previous study in humans (Devaine et al., 2014a), we modified the original *1-ToM* learning algorithm (Devaine et al., 2014b), such that it could identify stable biases in the behavioural tendencies of its opponent.

More generally, *k-ToM* agents ($k\geq2$) consider that their opponent is a $\kappa$*-ToM* agent with a lower ToM sophistication level (i.e.: $\kappa<k$). Importantly, the sophistication level $\kappa$ of *k-ToM*'s opponent has to be learned, in addition to the hidden states $x^{\kappa}$ that control the opponent's learning and decision making. The difficulty for a *k-ToM* agent is that she needs to consider different scenarios: each of her opponent's possible sophistication level $\kappa$ yields a specific probability $p^{op,\kappa}=s\circ v^{\kappa}\left(x^{\kappa}\right)$ that she will choose action $a^{op}=1$. The ensuing meta-Bayesian learning rule entails updating *k-ToM*'s uncertain belief about her opponent's sophistication level $\kappa$ and hidden states$x^{\kappa}$:

(S4)

where $\lambda_{t}^{k,\kappa}$ is *k-ToM*'s posterior probability that her opponent is $\kappa$*-ToM*, and $W^{\kappa}$ is the gradient of $v^{\kappa}$ with respect to the hidden states $x^{\kappa}$. Here, the mapping $v^{\kappa}$ is obtained by the recursive insertion of Equation S4 into the softmax policy, and $\tilde{v}^{\kappa}$ is defined implicitly in terms of the expectation operator, as follows:$E\left[s\circ v^{\kappa}\left(x_{t-1}^{k,\kappa}\right)\right]=s\circ\tilde{v}^{\kappa}\left(\mu_{t-1}^{k,\kappa},\Sigma_{t-1}^{k,\kappa}\right)$. Equation S4 is but a compact formulation of how the summary statistics ($\mu^{k}$, $\Sigma^{k}$ and $\lambda^{k}$) of *k-ToM*'s posterior distribution $q\left(x^{k},\kappa\right)$ evolve from trial to trial. Both Equations S2 and S4 have been derived using a variational approach to approximate Bayesian inference (Beal, 2003; Daunizeau et al., 2009; Friston et al., 2007). Although Equation S4 is slightly more complex than Equation S2, learning is still driven by a simple prediction error term. However, there is an interaction between the beliefs on the opponent's sophistication level and hidden states. For example, one can see that $\mu^{k,\kappa}$ and $\Sigma^{k,\kappa}$ are left unchanged if the $\kappa$-ToM scenario is unlikely, i.e. if $\lambda^{k,\kappa}\to0$. Also, $\lambda^{k,\kappa}$ increases in proportion to how likely was the opponent's last choice under the $\kappa$*-ToM* scenario $p_{t}^{op,\kappa}$, which depends upon $\mu^{k,\kappa}$ and $\Sigma^{k,\kappa}$.

When comparing different learning styles given observed trial-by-trial choice sequences in the main task, we made a distinction between "competitive" and "cooperative" *k-ToM* learning schemes (see main text). These differ in terms of subjective payoff tables that encode the implicit game rules. Under the competitive (factually correct) interpretation of the game, the payoff table is given in Table S3 below:

| Hider  Seeker | Left | Right |
| --- | --- | --- |
| Left | 1,0 | 0,1 |
| Right | 0,1 | 1,0 |

Table S3: Competitive payoff table. The primate plays the role of the seeker, the experimenter (zookeeper) is the hider.

Under the cooperative (factually incorrect) interpretation of the game, the payoff table is table is given in Table S4 below:

| Feeder  Seeker | Left | Right |
| --- | --- | --- |
| Left | 1,1 | 0,0 |
| Right | 0,0 | 1,1 |

Table S4: Cooperative payoff table. The experimenter (zookeeper) is not seen as a competitor but as a feeder.

Finally, note that *k-ToM* models do not differ in terms of the number of their free parameters. More precisely, *k-ToM*’s learning and decision rules are entirely specified by their prior volatility $\sigma^{k}$ (cf. Equations S2 and S4) and behavioural temperature $\beta$ (cf. Equation 1 of the main text).

This concludes the mathematical exposition of our meta-bayesian model of ToM agents. More details regarding the derivation of k-ToM algorithms can be found in (Devaine et al., 2014a, 2014b).

***Control task***

The observed perseverative behaviour estimated using Volterra analyses (see main text) may be partly non-instrumental (as originating from e.g., a limited flexibility to switch from one alternative action to the other one). We thus ran an additional control task that was aimed at quantifying the non-instrumental component of perseverative behaviour in each species. Critical in this control task is the fact that the reward's location was always visible to the animal, which results in the absence of choice incentive whenever both alternatives were equally rewarding (two trials out of three). We reasoned that in such trials, the individual's choice would mostly be driven by her (non-instrumental) perseverative tendency (if any). This then served as an out-of-sample estimate of baseline perseverative bias, which we include in all subsequent computational models of learning (see below).

The control task was administrated a few months after the main test (n=35; two lemurs, one mangabey and one orangutan could not been retested because they either changed zoo, or passed out in the elapsed time). Individuals were tested in a single session comprising few trials of habituation followed by 60 trials of the test phase (see below).

*Habituation phase.* The experimenter (zookeeper) presented his/her two hands open, one of which contained the reward (fruit) and the other some uninteresting item (straw or small stone). In contrast to the main task, individuals could actually observe what was in each hand before choosing. The position of the reward was randomized over trials. Similarly to the other experimental phases, the individual had to designate one hand by touching it and received what it contained (reward or straw). To complete the habituation phase, individuals had to choose correctly the hand containing the reward in five consecutive trials. (Note: for some individuals, only three such trials were performed for the habituation test, but a post-test revealed that these individuals too passed the five consecutive trials criterion). This ensured that the individuals were motivated by the rewards and understood that touching only one hand of the opponent would allow them to obtain the reward of that given hand.

*Test phase.* Here as well, the experimenter always presented the two hands open. The test phase comprised 60 trials with the following structure: in the first three trials, only one hand contained a reward ("single-hand" trial) as opposed to the other hand that contained a straw, like in the habituation phase. In the ensuing two trials, both hands of the zookeeper contained a reward ("both hands" trial). In such trials, there was no incentive to choose one hand over the other, beyond some potential default bias and/or (non-strategic) perseverative tendency. The sequence of observed responses was then decomposed using Volterra analyses, as for choices sequences in the main task (the opponent's action $a^{op}$ in Eq. 6 of the main text is now replaced by the visible reward location). The non-strategic perseverative component was measured using the magnitude of the Volterra kernel associated to one’s own previous actions.

***Bayesian Model Comparison***

First of all, all learning models described in the main manuscript were augmented with baseline perseverative Volterra kernels, which were estimated beforehand given data obtained in a specific control task (see supplementary materials), and included in the logistic likelihood function together with a potential (session-specific) bias. More precisely, the animals' sigmoidal decision rule (cf. Equation 1 of the main text) was modified as follows:

$P\left(a_{t}^{self}=1\right)=s\left(\frac{\Delta V}{\beta}+\omega^{0}+A^{self}\sum_{\tau} \exp\left(-\lambda^{self}\tau\right) \left(2a_{t-\tau}^{self}-1\right)\right)$ (S5)

where prior distributions for baseline perseverative parameters $A^{self}$ and $\lambda^{self}$ were set to their posterior distributions (as estimated from the control task). Note that is a conservative procedure, in the sense that evidence in favour of ToM-compatible learning styles demands more than a mere increase in perseverative behaviour. Nevertheless, it turned out that our model comparison results with and without including the baseline perseverative tendency are identical. Although this eventually mitigates the practical relevance of our control task, this supports the robustness of our model-based approach.

All learning models were inverted (given observed trial-by-trial choice sequences) using a variational-Laplace approach (Daunizeau et al., 2014; Friston et al., 2007). Bayesian log-model evidences $\ln p\left(a^{(i)}\left|m^{(j)}\right.\right)$ were then approximated using the so-called VB free-energy for each individual ($i$) and each model ($j$). One can then evaluate, for each individual, the posterior probability $pToM^{(i)}$ of exhibiting a ToM-compatible learning style, by normalizing the evidences and then summing over the family of ToM-compatible learning models, i.e. *Inf*, *1-ToM,* *2-ToM*, in both cooperative and competitive modes:

$pToM^{(i)}=\frac{\sum_{j\in\left\{\text{Inf,1-ToM,2-ToM}\right\}} p\left(a^{(i)}\left|m^{(j)}\right.\right)}{\sum_{j} p\left(a^{(i)}\left|m^{(j)}\right.\right)}$ (S6)

where the summation in the denominator of Equation S6 is over all models. This is essentially a specific instance of so-called family-inference (Penny et al., 2010). In the main manuscript, we report the average *pToM* over individuals belonging to the same species.

***Accounting for phylogeny when comparing primate species***

Comparing phenotypes of biological species is long known to be prone to subtle forms of statistical biases that arise from the fact that species are part of a hierarchically structured phylogeny (Felsenstein, 1985; Price, 1997). In brief, under standard (Brownian) genetic drift models, phylogenic relationships induce non-zero correlations between species' phenotypes (Felsenstein, 1988). In other terms, finding a given sample correlation across species may be less surprising under simple phylogenetic drift than under typical statistical null (chance) models.

This concern is relevant in our case, since we are drawing inferences based upon sample correlations between brain volume, group size and ToM sophistication (across species). Thus, we evaluated the distribution of such correlations under genetic drift on phylogenic trees using numerical simulations. First, we obtained the phylogenic tree of our sample of tested primate species from a public open database (Hinchliff et al., 2015). We then performed Monte-Carlo simulations to derive the distribution of Pearson correlations between pairs of dummy features. Note that we assumed equal branch length, which, in our case, inflates the effect of phylogenic structure (Kuhner and Felsenstein, 1994). Results are shown on Figure S3 below.


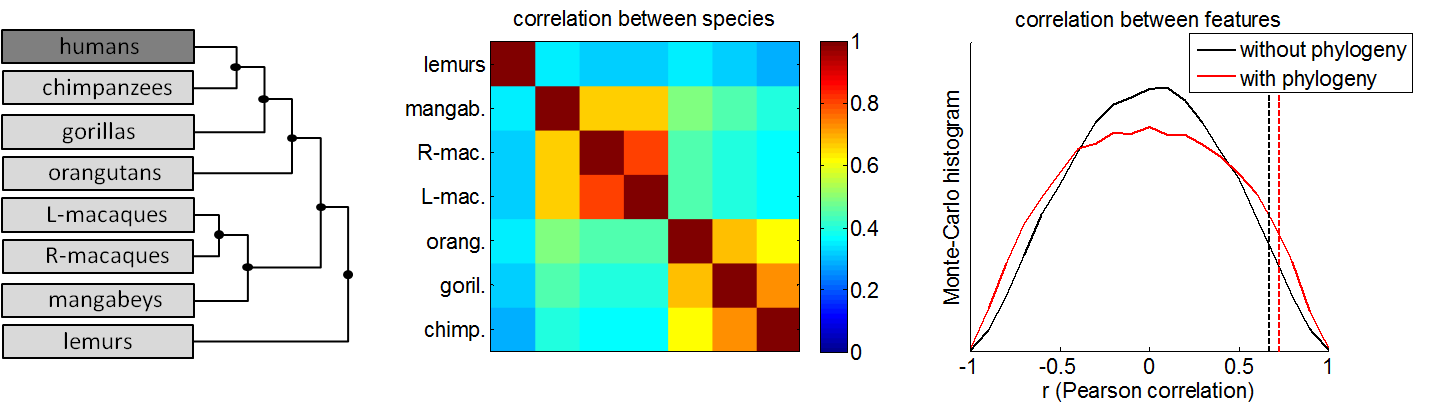


Figure S3: accounting for phylogeny in inter-species comparisons. Left: phylogenic tree showing the evolutionary relationships among tested primate species (and humans). Middle: phylogeny-induced correlation between species, obtained from Monte-Carlo simulations under to the standard evolutionary drift model. Right: Monte-Carlo histogram of correlation between dummy features with (red) and without (black) phylogenic structure. Dotted lines depict the ensuing p=0.05 threshold.

One can see that the hierarchical phylogenic tree structure induces a quasi block-diagonal between-species correlation matrix, with three clusters: (i) prosimians (lemurs), (ii) monkeys (mangabeys and macaques), and (iii) apes (chimpanzees, gorillas and orangutans). These subsets show both high intra-cluster and low inter-cluster correlations. Note that these clusters are visible as "grades" in the structure of correlation between brain volume and group size (cf. Figure 1 of the main manuscript). As expected, the distribution of between-features pairwise correlations under genetic drift on the tree is wider than under independence. In particular, the p=0.05 threshold for this subset of species is at r=0.73 (to be compared with r=0.67 under the "statistical null"). In the main text, we use these Monte-Carlo simulations to derive the statistical significance of pairwise sample correlations between species' sociobiological features.

***Additional Results***

***Performance***

*First level analysis*. At the subject level, we corrected the raw performance scores for the effects of session number and delay between sessions. Note that the session number had a significant positive effect on performance at the group level (p=.0001), whereas the effect of delay between session was not consistent across individuals (p=.23). This suggests that there may be a slight habituation effect in the main task, above and beyond within-games learning.

*Second level analysis.* At the group level, we tested whether inter-individual variability in performance scores could be predicted by our individual confounding variables (namely: Age, Sex, Rearing, see Table S2). Only Rearing showed a trend toward significance (R^2^=6.3%, p=.054). Note that, when correcting for multiple comparisons, the standard 5% significance threshold is alpha=0.017. This means that these nuisance variables could not confound our inter-species comparisons.

***Volterra analysis***

*First level analysis*. At the subject level, we corrected the estimated magnitudes of the Volterra kernels for the effect of session number and delay between sessions. Note that only the session number had a significant effect, and only on the amplitude for own previous actions at the group level (p=.0007), i.e. perseverative tendencies decreased with practice.

*Second level analysis.* At the group level, we tested whether inter-individual variability in Volterra kernels magnitudes could be predicted by our individual confounding variables. Age had a significant effect on both amplitudes (p<1e-5), such that older individuals exhibit a greater tendency to imitate and perseverate. In addition, females (p=.0003) and individuals who were raised by a human (p<1e-5) show a greater tendency to imitate their opponent.

***Control task***

We tested whether individual variables such as Age, Rearing, Sex and Bodyweight could predict inter-individual differences in non-strategic perseverative tendencies. We found that only Age had a significant effect (R^2^=29.2%, p=.004).

***Fit accuracy of learning models***

Although Volterra decompositions of trial-by-trial choice sequences are proper computational models of learning styles, they provide a reference point for our learning models in terms of fit accuracy. In brief, the average percentage of explained variance of Volterra decompositions was generally quite high, i.e. R^2^=71.7%. This is comparable to the most likely learning model, which yields a fit accuracy of R^2^=72.2%. Note that Volterra’s fit accuracy seemed to vary across opponent conditions (*RB*: R^2^=75%, *0-ToM*: R^2^=68%, *1-ToM*: R^2^=72%). When performing an ANOVA on Volterra’s fit accuracy for each individual in each condition, the effect of condition turned out to be significant (R^2^=32.1%, p<1e-5). The fit accuracy of the most likely learning model followed the same pattern.

***Model identifiability: confusion analyses.***

Different learning models may predict similar choice sequences, which may confuse Bayesian model selection. We thus quantified model identifiability using Monte-Carlo simulations performed under conditions similar to our experimental data analyses.

We first generated trial-by-trial choice sequences under each learning model (10 models, 60 trials per game, 3 opponents, 35 dummy subjects). For each simulated dataset (under a specific model), we performed a Bayesian Model Selection (across the 10 alternative learning models), and compared the winning model to the ground truth. For any given simulated model, we then measured the frequency with which each candidate model is eventually selected. Figure S4 below summarizes this analysis in terms of the so-called *confusion matrix*, which represents the expected frequency profile of selected models for each true model (in column).


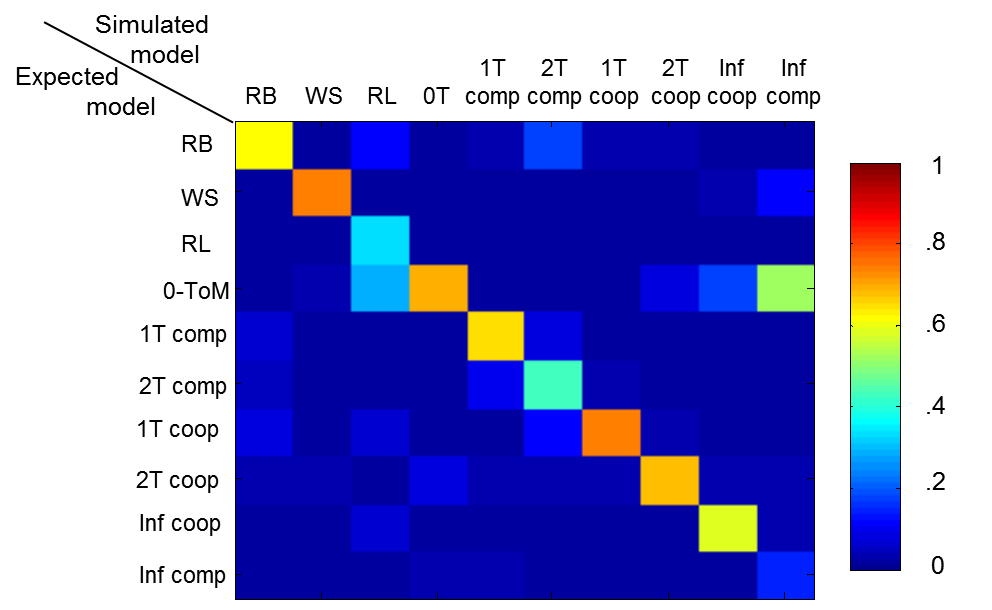


Figure S4: Confusion matrix. Each column of the confusion matrix shows the expected frequency of winning models, obtained when performing Bayesian model selection on data generated under the corresponding true model. Any non-diagonal element in this matrix signals a potential confusion between the inferred model and the true model.

Overall, one can see that the ten models are fairly identifiable under our experimental design. The only possible source of confusion is between the competitive *Influence* model and *0-ToM* (cf. tenth column of the confusion matrix: when simulating data under *Inf-comp*, model selection tends to favour *0-ToM*).

***Additional group-level (RFX) Bayesian model comparison***

Recall that we are looking for differences in learning styles that can be predicted from inter-species differences in terms of group size or ECV. Let us address this issue by first partitioning the species according to group size (using a median split) and then evaluating the evidence that the ToM model family is more frequent among species with big social groups. The result of this procedure is summarized on Figure S4-A below. Note that all the summary statistics reported here derive from state-of-the-art random-effect Bayesian model selection (RFX-BMS), which treats models as random effects that can differ across subjects. The ensuing statistical analysis estimate the underlying unknown frequency profile (over models) of the corresponding parent population (Penny et al., 2010; Rigoux et al., 2014; Stephan et al., 2009).


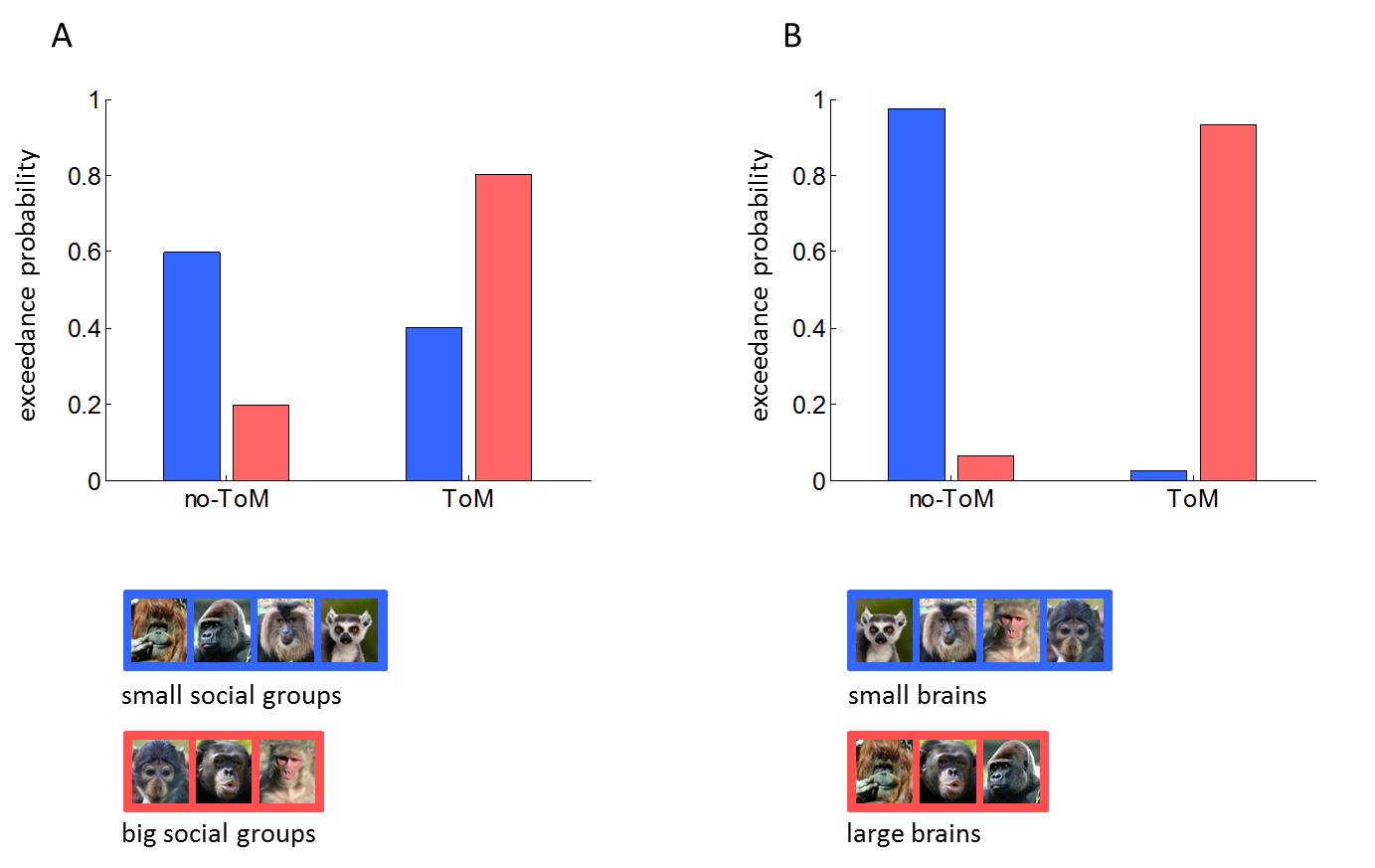


Figure S5: Bayesian model comparison results. The exceedance probability (y-axis) of each model families (left: no-ToM learning styles, right: ToM learning styles) is plotted as a function of species bi-partitions. Species are partitioned according to a median-split on either social group size (**A**: blue: species with small social groups, pink: species with large social groups) or ECV (**B**: blue: species with small brains, pink: species with large brains).

Figure S5-A reports the exceedance probability (EP) associated with each model family (ToM versus no-ToM), which corresponds to the posterior probability that each model family is the most frequent one within each species subgroup. One can see that the exceedance probability of the ToM family is EP=0.40 (estimated frequency = 47%) for species with small social groups, whereas EP=0.80 (estimated frequency = 59%) for species big social groups. In other terms, species with small (resp., big) social groups are slightly more (resp., less) likely to exhibit no-ToM learning styles than ToM learning styles. This seems to indicate that decreasing group size eventually compromises the expression of ToM-compatible learning styles. However, the direct experimental evidence for a difference in learning styles between the two subgroups of species is in fact quite weak. Using between-group RFX-BMS (Rigoux et al., 2014), we found that there is only a 0.78 probability that species with big social groups have evolved more sophisticated ToM than species with small social groups.

Now Figure S5-B reports the same family EPs, after having splat the species according to ECV. First, note that the average similarity of the two median splits is about 41% (chance level is 50% for uncorrelated features). However, one can see a much more marked difference: the exceedance probability of the ToM family is EP=0.03 (estimated frequency = 29%) for species with small brains, whereas EP=0.94 (estimated frequency = 67%) for species with large brains. This eventually results in stronger evidence for a difference in learning styles between the two subgroups of species: given observed choice sequences, there is a 0.99 probability that species with large brains have evolved more sophisticated ToM than species with small brains.

Note that, overall, this RFX-BMS approach proved much more sensitive than data-driven (Volterra) analyses of trial-by-trial choice sequences. This is partly because we reduced the statistical inference to dichotomous questions, by both using median splits on sociobiological features of primate species and partitioning our model space into two subfamilies (ToM versus non-ToM). In turn, we preserved the statistical power of our inference, at the cost of reducing our target experimental enquiry to a categorical question (namely: whether species that differ in terms of either group size or brain volume exhibit distinct learning styles in the context of repeated social interactions).

***References***

Beal, M.J. (2003). Variational algorithms for approximate Bayesian inference /. PhD Thesis.

Berger, U. (2007). Brown’s original fictitious play. J. Econ. Theory *135*, 572–578.

Daunizeau, J., Friston, K.J., and Kiebel, S.J. (2009). Variational Bayesian identification and prediction of stochastic nonlinear dynamic causal models. Phys. Nonlinear Phenom. *238*, 2089–2118.

Daunizeau, J., Adam, V., and Rigoux, L. (2014). VBA: A Probabilistic Treatment of Nonlinear Models for Neurobiological and Behavioural Data. PLoS Comput Biol *10*, e1003441.

Deaner, R.O., Isler, K., Burkart, J., and van Schaik, C. (2007). Overall Brain Size, and Not Encephalization Quotient, Best Predicts Cognitive Ability across Non-Human Primates. Brain. Behav. Evol. *70*, 115–124.

Devaine, M., Hollard, G., and Daunizeau, J. (2014a). The Social Bayesian Brain: Does Mentalizing Make a Difference When We Learn? PLoS Comput. Biol. *10*, e1003992.

Devaine, M., Hollard, G., and Daunizeau, J. (2014b). Theory of mind: did evolution fool us? PloS One *9*, e87619.

Dunbar, R.I.M. (1992). Neocortex size as a constraint on group size in primates. J. Hum. Evol. *22*, 469–493.

Dunbar, R.I.M. (1995). Neocortex size and group size in primates: a test of the hypothesis. J. Hum. Evol. *28*, 287–296.

Felsenstein, J. (1985). Phylogenies and the Comparative Method. Am. Nat. *125*, 1–15.

Felsenstein, J. (1988). Phylogenies and Quantitative Characters. Annu. Rev. Ecol. Syst. *19*, 445–471.

Finlay, B.L., and Darlington, R.B. (1995). Linked regularities in the development and evolution of mammalian brains. Science *268*, 1578–1584.

Friston, K., Mattout, J., Trujillo-Barreto, N., Ashburner, J., and Penny, W. (2007). Variational free energy and the Laplace approximation. NeuroImage *34*, 220–234.

Hinchliff, C.E., Smith, S.A., Allman, J.F., Burleigh, J.G., Chaudhary, R., Coghill, L.M., Crandall, K.A., Deng, J., Drew, B.T., Gazis, R., et al. (2015). Synthesis of phylogeny and taxonomy into a comprehensive tree of life. Proc. Natl. Acad. Sci. *112*, 12764–12769.

Hirata, S., Hirai, H., Nogami, E., Morimura, N., and Udono, T. (2017). Chimpanzee Down syndrome: a case study of trisomy 22 in a captive chimpanzee. Primates *58*, 267–273.

Isler, K., Christopher Kirk, E., Miller, J.M. a, Albrecht, G. a., Gelvin, B.R., and Martin, R.D. (2008). Endocranial volumes of primate species: scaling analyses using a comprehensive and reliable data set. J. Hum. Evol. *55*, 967–978.

Kuhner, M.K., and Felsenstein, J. (1994). A simulation comparison of phylogeny algorithms under equal and unequal evolutionary rates. Mol. Biol. Evol. *11*, 459–468.

Penny, W.D., Stephan, K.E., Daunizeau, J., Rosa, M.J., Friston, K.J., Schofield, T.M., and Leff, A.P. (2010). Comparing Families of Dynamic Causal Models. PLoS Comput Biol *6*, e1000709.

Price, T. (1997). Correlated evolution and independent contrasts. Philos. Trans. R. Soc. B Biol. Sci. *352*, 519–529.

Rigoux, L., Stephan, K.E., Friston, K.J., and Daunizeau, J. (2014). Bayesian model selection for group studies - revisited. NeuroImage *84*, 971–985.

van Schaik, C.P. (1999). The socioecology of fission-fusion sociality in Orangutans. Primates J. Primatol. *40*, 69–86.

van Schaik, C.P., and Burkart, J.M. (2011). Social learning and evolution: the cultural intelligence hypothesis. Philos. Trans. R. Soc. B Biol. Sci. *366*, 1008–1016.

Stephan, H., Frahm, H., and Baron, G. (1981). New and revised data on volumes of brain structures in insectivores and primates. Folia Primatol. Int. J. Primatol. *35*, 1–29.

Stephan, K.E., Penny, W.D., Daunizeau, J., Moran, R.J., and Friston, K.J. (2009). Bayesian model selection for group studies. NeuroImage *46*, 1004–1017.
