# Supplementary material for: Reading wild minds: A computational assay of Theory of Mind sophistication across seven primate species
Source: PLoS Comput Biol. 2017 Nov 7;13(11):e1005833. doi: 10.1371/journal.pcbi.1005833 (PMC5693450; doi:10.1371/journal.pcbi.1005833)
Supplement: S2 Text — (DOCX) [file pcbi.1005833.s002.docx]

***Table of references for species' group sizes***

| **Taxon** | **Group Size** | **References** |
| --- | --- | --- |
| *Cercocebus lunulatus* | 67 | Ehardt 1988 |
| *Cercocebus torquatus* | 21 | Mitani 1989 |
| *Cercocebus torquatus* | 18.5 | Smuts et al. 1987 |
| *Cercocebus torquatus* | 35 | Smuts et al. 1987 |
| *Cercocebus torquatus* | 37 | Rowe 1996 |
| *Cercocebus torquatus* | 105 | Range & Fischer 2004 |
| *Cercocebus torquatus* | 95 | Rowe 1996 |
| *Gorilla gorilla* | 11 | Parnell 2002 |
| *Gorilla gorilla* | 11 | Parnell 2002 |
| *Gorilla gorilla* | 13 | Parnell 2002 |
| *Gorilla gorilla* | 13 | Masi et al. 2009; Masi et al. 2015 |
| *Gorilla gorilla* | 14 | Parnell 2002 |
| *Gorilla gorilla* | 16 | Parnell 2002 |
| *Gorilla gorilla* | 2 | Parnell 2002 |
| *Gorilla gorilla* | 3 | Parnell 2002 |
| *Gorilla gorilla* | 4 | Parnell 2002 |
| *Gorilla gorilla* | 5 | Parnell 2002 |
| *Gorilla gorilla* | 6 | Parnell 2002 |
| *Gorilla gorilla* | 7 | Parnell 2002 |
| *Gorilla gorilla* | 7 | Parnell 2002 |
| *Gorilla gorilla* | 9 | Parnell 2002 |
| *Gorilla gorilla* | 9 | Parnell 2002 |
| *Gorilla gorilla* | 9.2 | Goldsmith 1993 |
| *Gorilla gorilla* | 8.2 | Gatti et al. 2004 |
| *Gorilla gorilla* | 5.5 | Gatti et al. 2004 |
| *Lemur catta* | 15.3 | Kappeler et al. 1996; Kappeler 1997; Atsalis 2000; Fietz et al. 2000; Sterling et al. 2006; Eberle et al. 2006; Lahann 2007; Rasoloharijaona et al. 2008: all from MacLean et al. 2009 |
| *Lemur catta* | 18 | Wrangham et al. 1993 |
| *Lemur catta* | 17 | Smuts et al. 1987 |
| *Lemur catta* | 17.2 | Sussman 1977; Howarth et al. 1986 from Dunbar 1991 |
| *Lemur catta* | 12 | Clutton-Brock & Harvey 1977 |
| *Lemur catta* | 17 | Rowe 1996 |
| *Lemur catta* | 15 | Jolly 1966; Oda 1996 |
| *Lemur catta* | 14 | Sussman et al. 1991 |
| *Lemur catta* | 11.5 | Gould et al. 2003 |
| *Lemur catta* | 13 | Sauther et al. 1993 |
| *Macaca mulatta* | 32 | Smuts et al. 1987 |
| *Macaca mulatta* | 32.8 | Smuts et al. 1987 |
| *Macaca mulatta* | 33.2 | Smuts et al. 1987 |
| *Macaca mulatta* | 39 | Smuts et al. 1987 |
| *Macaca mulatta* | 41 | Smuts et al. 1987 |
| *Macaca mulatta* | 41.3 | Smuts et al. 1987 |
| *Macaca mulatta* | 41.6 | Smuts et al. 1987 |
| *Macaca mulatta* | 47.7 | Smuts et al. 1987 |
| *Macaca mulatta* | 49.8 | Smuts et al. 1987 |
| *Macaca mulatta* | 32 | Teas et al. 1980 from Dunbar 1991 |
| *Macaca mulatta* | 21.5 | Clutton-Brock & Harvey 1977 |
| *Macaca mulatta* | 30 | Rowe 1996 |
| *Macaca mulatta* | 13 | Lindberg 1971 from Patterson et al. 2014 |
| *Macaca mulatta* | 13 | Makwana 1978 from Patterson et al. 2014 |
| *Macaca mulatta* | 18 | Melnick 1981 from Patterson et al. 2014 |
| *Macaca mulatta* | 19 | Makwana 1978 from Patterson et al. 2014 |
| *Macaca mulatta* | 21.5 | Southwick et al. 1965; Neville 1968; Lindberg 1971, 1977: all from Patterson et al. 2014 |
| *Macaca mulatta* | 25 | Melnick 1981; Pearl 1982 from Patterson et al. 2014 |
| *Macaca mulatta* | 29 | Melnick 1981 from Patterson et al. 2014 |
| *Macaca mulatta* | 31 | Makwana 1978 from Patterson et al. 2014 |
| *Macaca mulatta* | 38 | Makwana 1978 from Patterson et al. 2014 |
| *Macaca mulatta* | 39 | Teas et al. 1980 from Patterson et al. 2014 |
| *Macaca mulatta* | 50 | Melnick 1981 from Patterson et al. 2014 |
| *Macaca mulatta* | 51 | Melnick 1981 from Patterson et al. 2014 |
| *Macaca mulatta* | 58.8 | Lindburg 1977 from Patterson et al. 2014 |
| *Macaca mulatta* | 59 | Melnick 1981 from Patterson et al. 2014 |
| *Macaca mulatta* | 6 | Lindburg 1977 from Patterson et al. 2014 |
| *Macaca mulatta* | 6 | Makwana 1978 from Patterson et al. 2014 |
| *Macaca mulatta* | 65 | Melnick 1981 from Patterson et al. 2014 |
| *Macaca mulatta* | 25 | Sade 1972; Chapais 1986 from Kudo & Dunbar 2001 |
| *Macaca mulatta* | 12.3 | Chetry et al. 2003 |
| *Macaca mulatta* | 90 | Seth & Steh 1986 |
| *Macaca mulatta* | 41 | Hasan et al. 2013 |
| *Macaca mulatta* | 30 | Hasan et al. 2013 |
| *Macaca silenus* | 21 | Smuts et al. 1987 |
| *Macaca silenus* | 17 | Rowe 1996 |
| *Macaca silenus* | 16.3 | Singh et al. 2002 |
| *Macaca silenus* | 70 | Krishna et al. 2006 |
| *Macaca silenus* | 38 | Singh et al. 2001 |
| *Macaca silenus* | 22 | Kurup at al. 1993 |
| *Pan troglodytes* | 8.1 | Nishida et al. 1968 |
| *Pan troglodytes* | 40 | Itani at al. 1967 |
| *Pan troglodytes* | 28 | Wrangham et al. 1993 |
| *Pan troglodytes* | 50 | Smuts et al. 1987 |
| *Pan troglodytes* | 28 | Goodall 1965; Wrangham 1977 from Dunbar 1991 |
| *Pan troglodytes* | 27 | Clutton-Brock & Harvey 1977 |
| *Pan troglodytes* | 187 | Carlson et al. 2013 |
| *Pan troglodytes* | 21 | Shimada et al. 2009 |
| *Pan troglodytes* | 27.5 | Suzuki 1979 from Patterson et al. 2014 |
| *Pan troglodytes* | 28 | Clutton-Brock & Harvey 1977 |
| *Pan troglodytes* | 29 | Herbinger et al. 2001 from Patterson et al. 2014 |
| *Pan troglodytes* | 35 | Herbinger et al. 2001 |
| *Pan troglodytes* | 44 | Gomes et al. 2011 |
| *Pan troglodytes* | 51 | Potts et al. 2011 |
| *Pan troglodytes* | 76 | Laporte et al. 2010; |
| *Pan troglodytes* | 10.2 | Sugiyama 1988; Takahata 1990; Kawanaka 1990; Nishida et al. 1996 |
| *Pongo abelii* | 2 | Rowe 1996 |
| *Pongo abelii* | 1.7 | Van Schaik 1999 |
| *Pongo pygmaeus* | 1.8 | Rodman et al. 1986 from Wrangham et al. 1993 |
| *Pongo pygmaeus* | 1 | Mackinnon 1974 from Dunbar 1991 |
| *Pongo pygmaeus* | 2 | Rowe 1996 |

***References***

Atsalis, S. (2000), Spatial distribution and population composition of the brown mouse lemur (Microcebus rufus) in Ranomafana National Park, Madagascar, and its implications for social organization. Am. J. Primatol., 51.1: 61-78.

Carlson, B. A., Rothman, J. M. and Mitani, J. C. (2013), Diurnal Variation in Nutrients and Chimpanzee Foraging Behavior. Am. J. Primatol., 75: 342–349. doi:10.1002/ajp.22112

Chapais B. (1986) Why do adult male and female rhesus monkeys affiliate during the birth season? In: Rawlins RG, Kessler MJ, editors. The Cayo Santiago macaques: History, Behavior and Biology. Albany: State University of New York Press; 1986. pp. 173–200

Chapman, C. A. and Wrangham, R. W. (1993), Range use of the forest chimpanzees of Kibale: Implications for the understanding of chimpanzee social organization. Am. J. Primatol., 31: 263–273. doi:10.1002/ajp.1350310403

Charles H. Janson, Michele L. Goldsmith (1995); Predicting group size in primates: foraging costs and predation risks. Behav Ecol; 6 (3): 326-336. doi: 10.1093/beheco/6.3.326

Chetry, D., Medhi, R., Biswas, J., Das, D., Bhattacharjee, P.C., (2003). Nonhuman primates in the Namdapha national park, Arunachal Pradesh, India. Int. J. Primatol. 24, 383–388

Clutton-Brock, T. H. & Harvey, P. H. (1977). Primate ecology and social organization. J. Zool. 183, 1–39

Dunbar, R. I. M. (1991). Functional significance of social grooming in primates. Folia Primatol. 57, 121–131

Eberle, Manfred, and Peter M. Kappele (2006); Family insurance: kin selection and cooperative breeding in a solitary primate (Microcebus murinus). Behav. Ecol. Sociobiol. 60.4: 582-588.

Ehardt, C. L. (1988) Absence of strongly kin-preferential behavior by adult female sooty mangabeys (Cercocebus atys). Am. J. Phys. Anthropol. 76, 233-243

Fietz, Joanna, et al. (2000), High rates of extra-pair young in the pair-living fat-tailed dwarf lemur, Cheirogaleus medius. Behav. Ecol. Sociobiol. 49.1: 8-17.

Gatti, S., Levréro, F., Ménard, N. and Gautier-Hion, A. (2004), Population and group structure of western lowland gorillas (Gorilla gorilla gorilla) at Lokoué, Republic of Congo. Am. J. Primatol., 63: 111–123. doi:10.1002/ajp.20045

Gomes, C.M. & Boesch, C. (2011); Behav Ecol Sociobiol 65: 2183. doi:10.1007/s00265-011-1227-x

Gould, Lisa, R. W. Sussman, and Michelle L. Sauther. (2003); Demographic and life‐history patterns in a population of ring‐tailed lemurs (Lemur catta) at Beza Mahafaly Reserve, Madagascar: A 15‐year perspective. Am. J. Phys. Anthropol. 120.2: 182-194.

Hasan K, Aziz MA, Alam SM et al. (2013). Distribution of rhesus macaques (Macaca mulatta) in Bangladesh: inter-population variation in group size and composition. Primate Conserv; 26: 115–124

Herbinger, I., Boesch, C. & Rothe, H. (2001); Int. J. Primatol. 22: 143. doi:10.1023/A:1005663212997

Howarth, C. J., et al. (1986); Population ecology of the ring-tailed lemur, Lemur catta, and the white sifaka, Propithecus verreauxi verreauxi, at Berenty, Madagascar, 1981. Folia Primatologica 47.1: 39-48.

Itani, Junichiro, and Akira Suzuki (1967); The social unit of chimpanzees. Primates 8.4: 355-381.

Jolly, A. (1966). Lemur social behavior and primate intelligence. Science 153, 501–506

Kappeler, P. M. & Heymann, E. W. (1996). Nonconvergence in the evolution of primate life history and socio-ecology. Biol. J. Linn. Soc. 59, 297–326

Kappeler, Peter M. (1997); Determinants of primate social organization: comparative evidence and new insights from Malagasy lemurs. Biol. Rev. 72.1: 111-151.

Krishna, B. A., Singh, M., & Singh, M. (2006). Population dynamics of a group of lion-tailed macaques (Macaca silenus) inhabiting a rainforest fragment in the Western Ghats, India. Folia Primatologica, 77, 377–386

Kudo, H. & Dunbar, R. I. M. (2001). Neocortex size and social network size in primates. Anim. Behav. 62, 711–722

Kurup, G. U. & Kumar, A. (1993) Time budget and activity patterns of the lion-tailed macaque (Macaca silenus). Int. J. Primatol. 14, 27–39

Lahann, P. (2007); Feeding ecology and seed dispersal of sympatric cheirogaleid lemurs (Microcebus murinus, Cheirogaleus medius, Cheirogaleus major) in the littoral rainforest of south‐east Madagascar; J. Zool. 271.1: 88-98.

Lindberg, D.G. (1971) The rhesus monkey in north India: an ecological and behavioural study. In Primate Behavior: Developments in Field and Laboratory Research, Vol. 2, pp. 1-106. Ed. L. A. Rosenblum. Academic Press, New York

Lindburg, D.G. (1977). Feeding behaviour and diet of rhesus monkeys (Macaca mulatta) in a Siwalik forest in North India. In: T.H. Clayton-Brock (Ed.), Primate Ecology: Studies of Feeding and Ranging Behaviour in Lemurs. Monkeys and Apes. Academic Press, London, p. 233

Mackinnon, John R. (1974); In search of the red ape. Ballantine Books pub.

MacLean, E. L., Barrickman, N. L., Johnson, E. M. & Wall, C. E. (2009). Sociality, ecology, and relative brain size in lemurs. J. Hum. Evol. 56, 471–478

Makwana SC, (1978). Field ecology and behaviour of the rhesus macaque (Macaca mulatto.): I. Group composi- tion, home range, roosting sites, and foraging routes in the Asarori Forest. Primates 19:483-492

Marion N.C. Laporte, Klaus Zuberbühler (2010), Vocal greeting behaviour in wild chimpanzee females, Animal Behav. 80.3: 467-473.

Melnick, D. (1981). Microevolution in a population of Himalayan rhesus monkeys (Mucucu rnuluttu). Ph.D. diss., Yale University

Mitani, M. (1989). Cercocebus torquatus: adaptive feeding and ranging behaviors related to seasonal fluctuations of food resources in the tropical rain forest of south-western Cameroon. Primates 30, 307– 323

Neville M. K. (1968) Male leadership change in a free-ranging troop of Indian Rhesus monkey (Macaca Mulatta). Primates 9: 1–2. [VVI]

Nishida, Toshisada, and Kazuhiko Hosaka. (1996); Coalition strategies among adult male chimpanzees of the Mahale Mountains, Tanzania. Great ape societies: 114-134.

Nishida, Toshisada (1968); The social group of wild chimpanzees in the Mahali Mountains. Primates 9.3: 167-224.

Oda, Ryo (1996); Effects of contextual and social variables on contact call production in free-ranging ringtailed lemurs (Lemur catta). Int. J. Primatol. 17.2: 191-205.

Parnell, R. J. (2002), Group size and structure in western lowland gorillas (Gorilla gorilla gorilla) at Mbeli Bai, Republic of Congo. Am. J. Primatol., 56: 193–206. doi:10.1002/ajp.1074

Patterson, S. K., Sandel, A. A., Miller, J. A. & Mitani, J. C. (2014). Data quality and the comparative method: the case of primate group size. Int. J. Primatol. 35, 990–1003

Patterson, S. K., Sandel, A. A., Miller, J. A. & Mitani, J. C. (2014). Data quality and the comparative method: the case of primate group size. Int. J. Primatol. 35, 990–1003

Pearl, M. C (1982). Networks of social relations among Himalayan rhesus monkeys (Macaca mulatta). Unpublished doctoral dissertation, Yale University, New Haven, CT

Potts, K.B., Watts, D.P. & Wrangham, R.W. Int J Primatol (2011) 32: 669. doi:10.1007/s10764-011-9494-y

Range, F. & Fischer, J. (2004) Vocal repertoire of sooty mangabeys (Cercocebus torquatusatys) in the Tai National Park. Ethology 110, 301–321.

Rasoloharijaona, Solofonirina, Blanchard Randrianambinina, and Elke Zimmermann (2008); Sleeping site ecology in a rain‐forest dwelling nocturnal lemur (Lepilemur mustelinus): Implications for sociality and conservation. Am. J. Primatol. 70.3: 247-253.

Rowe, Noel. (1996); Pictorial guide to the living primates. Pogonias Press, 1996.

Sade D. S., (1972). Sociometrics of Macaca mulatta: I. Linkages and cliques in grooming matrices. Folia Primatologica 18, 196–223

Sauther, Michelle L., and Robert W. Sussman (1993); A New Interpretation of the Social Organization and Mating System of the Ringtaled Lemur (Lemur Catta). Lemur social systems and their ecological basis. Springer US, 1993. 111-121.

Seth PK , Seth S. (1986); Ecology and behaviour of rhesus monkeys in India . In: Else JG, Lee PC, editors. Primate ecology and conservation, Volume 2. Cambridge ( UK ): Cambridge Univ Pr. 89-103

Shimada M, K, Hayakawa S, Fujita S, Sugiyama Y, Saitou N (2009); Skewed Matrilineal Genetic Composition in a Small Wild Chimpanzee Community. Folia Primatol 80:19-32

Singh M, Kumara HN, Kumar MA, Sharma AK. (2001). Behavioural responses of lion-tailed macaques (Macaca silenus) to a changing habitat in a tropical rainforest fragment in the Western Ghats, India. Folia Primatol 72:278–291

Singh, M., Kumar, M.A., Kumara, H.N., Sharma, A.K., Kaumanns, W., (2002). Distribution, population structure, and conservation of lion-tailed macaques (Macaca silenus) in the Anaimalai Hills, Western Ghats, India. American Journal of Primatology 57

Smuts, B. B., Cheney, D. L., Seyfarth, R. M. & Wrangham, R. W. Primate Societies (Univ. Chicago Press, 1987)

Sterling, Eleanor J., and Erin E. McCreless (2006); Adaptations in the aye-aye: a review. Lemurs. Springer US, 2006. 159-184.

Sugiyama, Y. Int J Primatol (1988) 9: 393. doi:10.1007/BF02736216

Sussman, R. W. (1991), Demography and social organization of free-ranging Lemur catta in the Beza Mahafaly Reserve, Madagascar. Am. J. Phys. Anthropol., 84: 43–58. doi:10.1002/ajpa.1330840105

Suzuki, A. (1979); The variation and adaptation of social groups of chimpanzees and black and white colobus monkeys. Primate Ecology and Human Origins. Garland, New York (1979): 153-173.

Takahata, Yukio (1990); Social relationships among adult males. The chimpanzees of the Mahale Mountains: sexual and life history strategies. University of Tokyo Press, Tokyo (1990): 133-148.

Teas, J., Richie, T., Taylor, H. & Southwick, C. H. (1980). Population patterns and behavioural ecology or rhesus monkeys (Macca mulatta) in Nepal. In: The Maquaces (Ed. by Lindburg, D. G.), pp. 247-262. New York: Van Nostrand Reinhold

Van Schaik, Carel P. (1999); The socioecology of fission-fusion sociality in orangutans. Primates 40.1: 69-86.

Wrangham, R. W., Gittleman, J. L. & Chapman, C. A. (1993); Constraints on group size in primates and carnivores: population density and day-range as assays of exploitation competition. Behav. Ecol. Sociobiol. 32, 199–209.
